# How the Physicochemical Properties of Manufactured Nanomaterials Affect Their Performance in Dispersion and Their Applications in Biomedicine: A Review

**DOI:** 10.3390/nano12030552

**Published:** 2022-02-06

**Authors:** Spiros H. Anastasiadis, Kiriaki Chrissopoulou, Emmanuel Stratakis, Paraskevi Kavatzikidou, Georgia Kaklamani, Anthi Ranella

**Affiliations:** 1 Institute of Electronic Structure and Laser, Foundation for Research and Technology-Hellas, N. Plastira 100, 700 13 Heraklion, Crete, Greece; kiki@iesl.forth.gr (K.C.); stratak@iesl.forth.gr (E.S.); ekavatzi@iesl.forth.gr (P.K.); georgina@iesl.forth.gr (G.K.); ranthi@iesl.forth.gr (A.R.); 2 Department of Chemistry, University of Crete, 700 13 Heraklion, Crete, Greece; 3 Department of Physics, University of Crete, 700 13 Heraklion, Crete, Greece

**Keywords:** physical/chemical characteristics, functionality, nanoparticles, nanomaterials

## Abstract

The growth in novel synthesis methods and in the range of possible applications has led to the development of a large variety of manufactured nanomaterials (MNMs), which can, in principle, come into close contact with humans and be dispersed in the environment. The nanomaterials interact with the surrounding environment, this being either the proteins and/or cells in a biological medium or the matrix constituent in a dispersion or composite, and an interface is formed whose properties depend on the physicochemical interactions and on colloidal forces. The development of predictive relationships between the characteristics of individual MNMs and their potential practical use critically depends on how the key parameters of MNMs, such as the size, shape, surface chemistry, surface charge, surface coating, etc., affect the behavior in a test medium. This relationship between the biophysicochemical properties of the MNMs and their practical use is defined as their functionality; understanding this relationship is very important for the safe use of these nanomaterials. In this mini review, we attempt to identify the key parameters of nanomaterials and establish a relationship between these and the main MNM functionalities, which would play an important role in the safe design of MNMs; thus, reducing the possible health and environmental risks early on in the innovation process, when the functionality of a nanomaterial and its toxicity/safety will be taken into account in an integrated way. This review aims to contribute to a decision tree strategy for the optimum design of safe nanomaterials, by going beyond the compromise between functionality and safety.

## 1. Introduction

The rapid expansion of nanotechnology and of the related synthesis and analysis tools has led to a significant increase of the variety of manufactured nanomaterials (MNMs) and of their range of applications. The term MNMs signifies *intentionally manufactured* materials ‘containing particles, in an unbound state or as an aggregate or as an agglomerate and where, for 50% or more of the particles in the number size distribution, one or more external dimensions is in the size range 1–100 nm’. Moreover, fullerene, graphene, and carbon nanotubes with minimum diameters below 1 nm are included as well. The definition of ‘nanomaterial’ was given in 2011 in a European Commission recommendation [1], where nanomaterials were also categorized as natural, incidental, or manufactured. This expansion in the application of MNMs has significantly increased the probability of them coming in contact with humans, the environment, and, in general, the Earth system [2]. It is, therefore, of great importance to identify all probable deleterious effects that MNMs may have on both human health and the environment, early on in the innovation process. A first step towards achieving this objective is to be able to link the physicochemical characteristics of the manufactured nanomaterials to their functionality. At the same time, much research work is still required to both, advance our knowledge on the physicochemical characterization of MNMs, and to explore on how these characteristics and the resulting properties affect their potential to induce toxicity in different receptors, as well as determine their ultimate fate [3]. The importance of lacking the right correlations regarding how physicochemical characteristics influence the fate of manufactured nanomaterials has been emphasized in reports on the life-cycle assessment of these MNMs [4]. Moreover, correlating the physico-chemical characteristics of MNMs and their extensive (eco)toxicological assessment would allow the application of grouping and read-across methodological approaches, which have been extensively used for chemicals in general and, based on the similarity between substances and their behavior, could be used to fill data gaps for other MNMs, without performing additional effort, and time, consuming testing [5]. One should also refer here to a classic book by Otterstedt and Brandreth [6], which deals with the chemical and physical principles of methods for the preparation of MNMs, as well as with the description of their surface and of the methods of its modification. The applications of small particle technology are also demonstrated, considering how to make technically important materials.

When any type of a nanomaterial interacts with a biological medium, which can consist of proteins, membranes, cells, organelles, and nucleic acids, various kinds of nanoparticle/biological interfaces are established, where the behavior is governed by the relevant biophysicochemical interactions, as well as by colloidal forces. These kinds of interactions can lead to the formation of protein coronas on the surface of the nanomaterials, wrapping of nanoparticles by membranes, intracellular uptake, and biocatalytic processes that could potentially have biocompatible or bio-antagonistic outcomes. At the same time, the nanomaterial surface may suffer phase transformations, restructuring, and/or dissolution, due to the presence of the biomolecules and the dispersing liquid medium. Being able to understand the structure and the behavior at such interfaces would allow predictive relationships between structure and activity to be developed, which will be governed by the nanomaterial characteristics, such as size, shape, roughness, surface chemistry, and surface coatings. Such knowledge will be imperative for the safe use of the nanomaterials [7].

Our main objective has been to identify, classify, and prioritize the physicochemical characteristics of nanomaterials in relationship to their functionalities, in order to demonstrate the interrelationship between these different functionalities and to illustrate the effect of the physicochemical properties on the MNM performance. The number of different nanoparticles, their properties, and their practical uses are vast, as are their different physicochemical properties and the resulting biophysicochemical interactions at the respective interfaces. Thus, it is not possible to discuss all of them in sufficient detail. In this work, we present a short review of how specific key parameters of manufactured nanomaterials affect some of these functionalities, except toxicity, which is, by itself, a huge field of research. Key parameters relative to geometry (particle size, particle shape, and aspect ratio), chemistry (composition, surface groups, surface charge), crystallinity, morphology (topology, roughness, porosity, and surface area), surface functionalization (surface coatings, reactivity, and stability), and test media (mostly aqueous) are discussed in relation to MNM functionalities. These functionalities are discussed in terms of two groups: performance or properties, on the one hand, and applications, on the other. In the properties/performance functionalities we have included dispersion ability in aqueous media, solubility/dissolution characteristics, and hydrophobicity/hydrophilicity, which are directly affected by the physicochemical characteristics of the prepared nanomaterials, but, at the same time, they can have an effect on the activity and the practical uses of the MNMs. In the second functionality group, we have included applications such as the cellular uptake of the MNMs, as well as their optical, electronic, magnetic, and catalytic properties. Since the number of MNMs is vast, we tried to focus our report mainly on certain MNMs that are more frequently encountered in contact with humans, such as titania (TiO_2_), silica (SiO_2_), zinc oxide (ZnO), cerium oxide (CeO_2_), iron oxide (Fe_3_O_4_), barium sulfate (BaSO_4_), cadmium selenide (CdSe) quantum dots, gold (Au), silver (Ag), and various carbon nanomaterials such as carbon nanotubes (CNTs), graphene, graphene oxide, and reduced graphene oxide. It is noted that carbon black (nano)materials, which are broadly used in tires, are not discussed in this mini review, mostly because this is a very broad area, where various grades of carbon black are used, often with a non-disclosed primary particle size distribution, as well as different sizes and structures of aggregates [8].

One should note that being able to understand such interrelationships will allow engineering the MNMs so that one can maximize the benefits for functionality, while reducing the risks to human health and/or the environment and, moreover, being able to achieve this at an early phase of the innovation process. This would enable the consideration of safety aspects for humans and the environment early on in the process of designing a new product, so as to minimize or, even, eliminate the risks of adverse effects during its life cycle, which includes synthesis, storage, use, maintenance, and decommission.

## 2. How the Key Parameters Affect Functionalities with Respect to Performance

### 2.1. Dispersion Ability

The state of dispersion of nanomaterials in the different dispersing media is a very important characteristic of nanoparticulates; yet this state is very challenging to quantify, since dispersion is a very complicated (and little understood) process [9,10]. Controlling the dispersion of fine particles and preventing the formation of uncontrollable aggregates can lead to materials with improved properties [11]. The aggregation of nanomaterials depends both on the particle characteristics (e.g., size, shape, concentration, surface charge, and surface roughness) and on the physicochemical properties of the media (e.g., ionic strength, pH, and/or presence of organic macromolecules) [12]. In the absence of a surface coating, the aggregation/disaggregation of nanomaterials is mainly controlled by the intrinsic properties of the particles, such as size and zeta (ζ)-potential, as well as by the ionic strength of the solutions, as described by the DLVO theory proposed by Derjaguin, Landau, Verwey, and Overbeek [13,14].

Nanoparticles tend to agglomerate immediately in cell culture media. Thus, the effects of the various biological dispersion media on the state of aggregation of the nanoparticles has been extensively investigated in the literature, especially since these are critical in evaluating and interpreting the toxicological assay results [15]. At the same time, when natural organic matter (NOM) is present, it usually increases the stability of the nanoparticles in water [12,16], whereas chemical surfactants, serum, and/or proteins are frequently used to improve the dispersion and stabilization of nanoparticles [17].

#### 2.1.1. Dispersibility of Metal and Metal Oxide Nanomaterials

**Figure 1:**
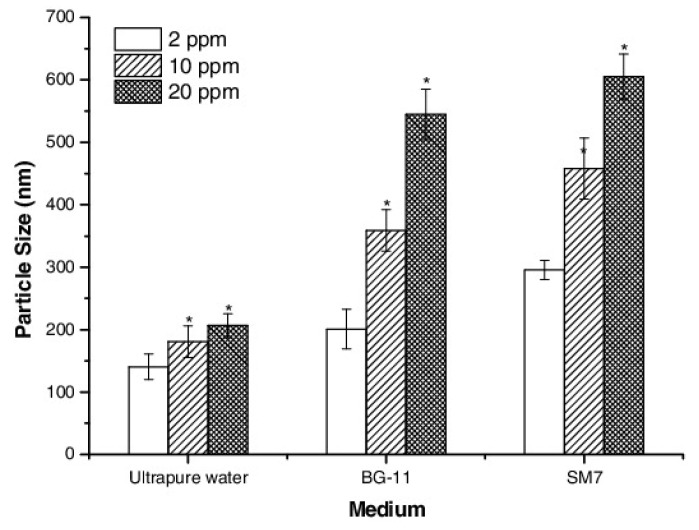
Dynamic light scattering (DLS) results for the size of TiO_2_ agglomerates as a function of TiO_2_ concentration in water, in freshwater microalgae cultured in Blue-Green medium (BG-11), and in daphnia magna cultured in simplified Elendt M7 medium (SM7). * denotes statistical differences from the control [18].

Titanium oxide (TiO_2_) nanoparticles are widely utilized in many different applications, for example, in cosmetics and sunscreen products; nevertheless, they may be toxic in certain cases and/or certain environments or aggregate in different culture media and, thus, the investigation of the degree of their dispersion is critical. Ultrapure water was found to disperse TiO_2_ better than freshwater microalgae and daphnia aquatic culture media (Figure 1). The hydrodynamic size of the nanoparticles was found to slightly depend on concentration in the former case; whereas, the effect was significantly larger for the latter [18].

In contrast, attempts to disperse TiO_2_ nanoparticles in water, even under strong sonication, led to sizes bigger than the hydrodynamic radius of the primary nanoparticles, indicating that the TiO_2_ sample consists of a certain number of strong aggregates that cannot be broken down easily, even when ultra-sonication is utilized [19]; the dispersion state was much poorer when different cell culture media were used in the absence of any dispersing agents. Bovine serum albumin (BSA) greatly improved the dispersion of nanoparticles in many culture media, with the observed differences attributed to the different protein–nanoparticle interactions in the media. On the other hand, fetal bovine serum (FBS) was found to be the best agent for dispersing and stabilizing TiO_2_ nanoparticles, due to the various proteins it comprises, which function in a synergistic manner. When rat and mouse bronchoalveolar lavage fluid (BALF) was used as a suspension medium, it was found to considerably reduce the aggregation of TiO_2_ (as well as ultrafine and fine carbon black); whereas, the use of phosphate buffered saline (PBS) containing protein or DPPC alone, in similar concentrations to those found in BALF, was not successful in satisfactorily dispersing the particles [20]. In another study, similar nanoparticle size distributions were measured in water without and with bovine serum; whereby, further dilution in Roswell Park Memorial Institute (RPMI) cell culture medium resulted in significant aggregation [21].

The type of biological medium in the presence of serum, together with the size of the nanoparticles, were found to affect the aggregation behavior of SiO_2_ nanoparticles; their primary size was measured when dispersed in water or media without serum [15]. In contrast to SiO_2_ nanoparticles, which showed a significant dependence of their measured size on the dispersion medium and/or on the presence of a protein, the size of poly(acrylic acid)-coated cobalt ferrite nanoparticles was found to be insensitive to the medium [22]. Moreover, the size of magnetic iron oxide nanoaggregates can be kept low, due to their stabilization via adsorption of FBS proteins [23], whereas the same protein reduces the agglomeration of zinc oxide nanoparticles [24], similarly to its effect on the dispersion of TiO_2_ nanoparticles mentioned above [19]. For hydroxyapatite nanomaterials, the nanoparticle size decreased with increasing FBS concentration in conjunction with stirring, which provides the necessary steric and electrostatic repulsion to overcome the attractive van der Waals forces and preserve the dispersion stability for a long period [25]. Fetal calf serum (FCS) was not successful in supplementing the dispersion of Au nanoparticles of different sizes in deionized water (DI); whereas, when it was used in Dulbecco’s modified eagle’s medium (DMEM), it led to the formation of complexes [26].

Temporarily stable small aggregates were formed when Al_2_O_3_ nanoparticles were dispersed either in deionized water (DI) or in ethylene glycol [27], whereas CeO_2_ nanoparticles formed a more stable dispersion only in water, in comparison to a fish medium in which sedimentation was clearly observed [28]. However, in both cases dispersions of small aggregates and not of primary particles were obtained. Moreover, citrate capped silver (Ag) nanoparticles in aqueous matrices were found to aggregate more pronouncedly in salty sea water compared to lake fresh water, due to the presence of natural organic matter (NOM), i.e., alginate humic and fulvic acids, and the low ionic strength of fresh water when compared to sea water [29]. The measured hydrodynamic radii were also found to decrease with increasing pH.

The dispersion of Ag nanoparticles, their aggregation, as well as the size of these aggregates and their stability were found to be very different in different organic solvents [30]. Ag (80 nm), hydrocarbon-coated Ag (15 nm and 25 nm), and polysaccharide-coated Ag (10, 25–30 and 80 nm) showed a similar tendency since they agglomerate at almost the same size when they are dispersed in water or media with serum; when media without serum were utilized, higher agglomeration sizes were obtained [31]. At the same time, the dispersion of metal and metal oxide nanoparticles, such as Al, Al_2_O_3_, Cu, SiO_2_, TiO_2_, and Ag, was investigated in water, cell culture media (RPMI-1640) only, and/or cell culture media with serum [31]. In the majority of cases, media without serum exhibited the worst dispersing ability, irrespectively of the kind of nanoparticles, their size, and/or their coating; whereas, in general, the media with serum were the best, differences in the final sizes were observed depending on the kind of nanoparticles in water. Moreover, the effect of the particle primary size on the agglomeration was very weak, if not absent. TiO_2_ nanoparticles exhibited high agglomeration, whereas SiO_2_ particles and SiO_2_-coated fluorophores (35, 51, and 110 nm) were the only nanoparticles that were dispersed in a way whereby the size of the primary particles could be measured. The dispersibility of CuO and ZnO nanoparticles was tested in different mineral and complex test environments, as well as its relationship with toxicity towards selected environmentally relevant test organisms and mammalian cells in vitro [32]. Both, CuO and ZnO nanoparticles were very unstable and sedimentation was observed. A considerably high degree of agglomeration/sedimentation was observed in the mineral media that are used for key regulatory ecotoxicological assays (crustaceans, algae). On the contrary, the components of the complex test media (test environment with organic components) were found to be critical in dispersing the nanoparticles and preventing their sedimentation.

The crystallinity and the primary size of nanoparticles are also factors that influence their dispersibility. In the case of TiO_2_, 100% anatase, 61–39% rutile-to-anatase, 40–60% rutile-to-anatase, as well as completely amorphous TiO_2_ nanoparticles were evaluated in water and in media with and without serum [31]. The amorphous TiO_2_ showed a high degree of agglomeration in all three suspending media, whereas the other TiO_2_ particles showed slightly smaller aggregates in water, and only the 61% rutile TiO_2_ showed a significant decrease in media with serum. The 61% rutile titania also exhibited the highest values of zeta-potential. Moreover, when the size of the TiO_2_ nanoparticles was studied utilizing nominally 5, 10, 16, 50, and 100 nm nanoparticles, a high agglomeration was obtained in all three media, except the 10 nm TiO_2_ in water. The effect of nanoparticle size on dispersibility has also been investigated with Au nanoparticles of 10, 50, 100, and 250 nm in aqueous suspensions diluted in phosphate buffered saline (PBS), to obtain a physiological solution [33]. The coexistence of agglomerates consisting of loosely arranged nanoparticles with individual ones was observed in all dispersions, except for the one of the largest nanoparticles, where there was not any obvious clustering. Particle shape also influences the electrostatic and steric repulsive forces, which are much stronger between two plate-like particles than between two spherical particles of the same volume, due to the much larger interaction surface between the plate-like particles [34].

#### 2.1.2. Dispersibility of Carbon Nanomaterials

**Figure 2:**
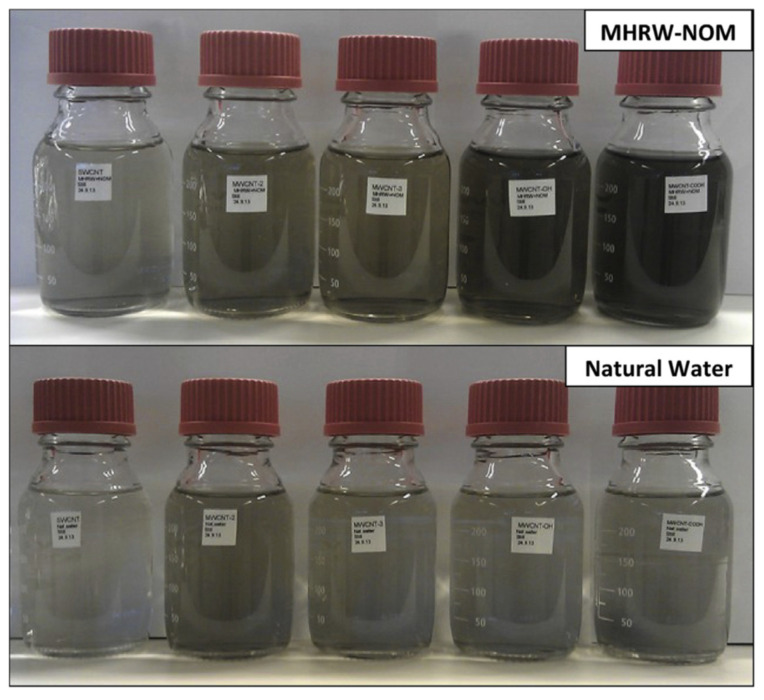
Different dispersibilities among CNT types and between different media, illustrated by CNT dispersions in MHRW-NOM (**top**) and natural water (**bottom**). From the left: SWCNT, MWCNT-15, MWCNT-30, MWCNT-OH, and MWCNT-COOH. (Reprinted with permission from ref. [16]. Copyright 2018 Elsevier).

More so than the dispersion of inorganic, metallic, or metal oxide nanoparticles, the prevention of aggregation in carbon nanomaterials is of utmost importance, since their agglomeration may hinder the realization of their excellent properties. Enhanced dispersion and stabilization of carbon nanomaterials (CNMs), such as graphene oxide, graphene, carbon nanotubes, and fullerenes, especially in water, is a critical challenge, because of their tendency to aggregate, particularly in aqueous systems, due to significant van der Waals attractions and their specific hydrophobic interactions [35]. It is both the physicochemical properties of the carbon nanomaterials and the properties of the dispersion medium that influence the dispersion stability, which is further enhanced in aqueous media with NOM, due to the enhanced interactions assisted by the CNMs hydrophobic surfaces. Both single- and multi-wall carbon nanotubes (SWCNTs and MWCNTs) were found to disperse better in media with NOM than in natural water (Figure 2); nevertheless, functionalization of the MWCNTs can improve the dispersion and lead to differences among the different media [16]. The presence of proteins, lipids, or protein/lipid components is crucial for the dispersion of carbon nanomaterials such as fullerenes and single- and multi-wall carbon nanotubes in various media as well [36], whereas vehicles lacking lipids or proteins lead to the formation of the largest agglomerates.

Aqueous suspensions of nanosilver, nanocopper, and fullerenes (C60) [37] were prepared in deionized water and in filtered natural river water to examine the effect of different concentrations of dissolved organic carbon (DOC) and different ionic strengths of the solutions; it was found that water chemistry influences both the suspension/solubility of the nanomaterials, as well as their particle size distributions. The dispersion of carbon nanotubes and carbon black was studied in water, in cell culture media (RPMI-1640), and/or in cell culture media in the presence of serum [31]. SWCNTs, MWCNT-COOHs, and CNTs formed aggregates in deionized water, whereas carbon black showed a large range of agglomeration sizes (the smaller found in water) depending on the solvent used. Stable aqueous dispersions of fullerenes, C60 and C70, were prepared in a different study by injecting a saturated suspension of fullerenes in tetrahydofuran (THF) into water and subsequently removing the THF by purging with nitrogen gas [38]. Fullerenes were dispersed as monodisperse clusters in water, and the obtained dispersions exhibited excellent colloidal stability, despite the absence of any stabilizing agent. This was attributed to the negatively charged surfaces that led to significant electrostatic repulsion and, thus, caused the stability of the dispersions.

#### 2.1.3. Surface Modification and Dispersibility

One of the most widely used methods to improve the dispersion stability of nanoparticles is their surface modification [39]. This necessitates a different designing of the surface structure, depending on the type of nanoparticle, as well as of the dispersing liquid media. Colloidal stability can be achieved by the adsorption, grafting, and/or coating of polymers, surfactants, and charged or biological molecules [34,40,41] that will provide electrostatic or steric repulsion between nanoparticles, thus, avoiding their agglomeration. In certain media, in order for a good dispersion of nanoparticles to be achieved, either a formulation with dispersants (usually amphiphilic molecules) or surface modification is requisite. For the latter case, the best functioning grafting molecules depend strongly on the size of the nanoparticle, with surfactants working better for small nanoparticles (<10–50 nm), whereas alkoxysilanes work better for larger ones (>50 nm) [42].

One of the simplest surface modification methods for improving dispersion stability is the adsorption of a polymeric dispersant on the surface of the nanoparticles; this methodology was presented in a comprehensive review [39]. Cationic or anionic polymer dispersants are commonly utilized to disperse nanoparticles, in either aqueous media or in organic solvents with high polarity; the polymer chains generate the steric repulsive force and increase the surface charge. Poly(acrylic acid) (PAA), sodium salts of PAA, as well as copolymers of acrylic acid and maleic acid are common anionic polymeric surfactants utilized to disperse oxide nanoparticles, such as TiO_2_, BaTiO_3_, Fe_2_O_3_, MgO, and Al_2_O_3_, whereas polyethyleneimine, PEI, is a commonly used cationic surfactant. The adsorption of the surfactants on the nanoparticles and the resulting range and magnitude of the repulsive force are influenced by a combination of various parameters, such as the suspension pH and solid fraction, the molecular weight of the polymer and its degree of dissociation, as well as the nanoparticle surface charge and its particle size. It was found that polymeric surfactants with a high molecular weight diffuse more difficultly around small nanoparticles and, thus, they cannot efficiently adsorb on their surface, failing to improve the dispersion stability of the suspension. Moreover, the dispersion stability can be affected by the surfactant structure. For example, for a polymer dispersant with a hydrophilic and a hydrophobic group, the ratio of the hydrophilic and hydrophobic sites controls the loop-train structure of the polymer adsorbed onto the particle surface, thus, affecting the dispersant ability. Copolymers possessing hydrophilic and hydrophobic segments are often utilized as anionic surfactants, to assist the dispersion of hydrophobic nanoparticles, such as SiC, CNTs, and coal, in aqueous media, since they can adsorb on the surface via their hydrophobic segments. Moreover, an aromatic monomer, such as styrene, can further improve the adsorption via both hydrophobic and π-π interactions. At the same time, the hydrophilic parts provide the necessary compatibility with the aqueous dispersing media and create an effective repulsive steric force. Cationic polymers, such as PEI, can also be utilized to enhance the dispersion of hydrophobic particles, such as SiC and CNTs, in aqueous media. Another method to improve the degree of dispersion of nanoparticles in various liquids is chemical modification of their surface. Silane coupling agents are utilized to alter the surfaces of oxide nanoparticles via the introduction of various reactive groups, such as epoxides, amines, and vinyls, on the particle surface and the subsequent grafting-from or grafting-to of polymers onto the surface. It is noted that neutral polymers, such as poly(ethylene oxide) or dextran, can also be employed as stealth coating agents to improve the colloidal stability and pass through physiological barriers; the most common cell targeting agents are proteins, enzymes, antibodies, or nucleotides [43].

Adsorption of certain surfactants on the outer or the inner surface of halloysite nanotubes has been utilized to increase their dispersibility, either in water or in organic solvents. At the same time, covalent or non-covalent functionalization of boron nitride nanotubes creates homogeneous dispersions in aqueous and organic media [44]. The dispersion stability of copper oxide (CuO) was investigated in different media, in their pristine form and when modified by four different stabilizing agents that gave them a negative (sodium ascorbate, ASC, and sodium citrate, CIT), a positive (polyethylenimine, PEI), or a neutral (polyvinylpyrrolidone, PVP) surface charge. The results showed that, in media with low ionic strength, the first two materials improved the dispersion by improving the repulsive potential, due to the negative charge, where PEI had the most significant effect, since it provides both electrostatic and steric stabilization, due to the positive charge and its polymeric nature, respectively. Amino acid and protein-rich media, however, control the stability irrespectively of the coating molecule [45].

An optimal concentration of sodium dodecylbenzene sulfonate (SDBS) was attained in the case of CuO and Al_2_O_3_ particles in deionized water, based on the reduction of their hydrodynamic radii that led to a concurrent decrease of viscosity and increase of thermal conductivity [46]. At the same time, SDBS and cetyltrimethylammonium bromide (CTAB) were utilized at low concentration and at exactly the critical micelle concentration (CMC) to assist the Al_2_O_3_ nanoparticle dispersion [47]. SDBS at CMC showed the best dispersion, because of the positive surface charge of alumina in the aqueous medium and its strong affinity for anionic groups, in contrast to CTAB, which, being a cationic surfactant, is repelled by the positively charged alumina surfaces. Similarly, SDBS was found to provide a better stability of Al_2_O_3_ nanoparticles than CTAB or SDS, whose performance was rather poor. In the former case, the measured hydrodynamic radius of the nanoparticles was approximately that of the primary ones, taking into account the size of the additional surfactant layer [48]. Beyond the stabilization in a simple nanofluid, SDBS shows a better and longer stabilization, lower hydrodynamic size, and narrower polydispersity than SDS, even for nanohybrid TiO_2_-Ag nanoparticles [49]. In a similar way, a certain concentration of PVP surfactants in a Al_2_O_3_/ethylene glycol nanofluid provides the most stable dispersions for long durations, due to the polymeric chain interactions, in contrast to the case when SDS is used, where a fast sedimentation is observed [50].

**Figure 3:**
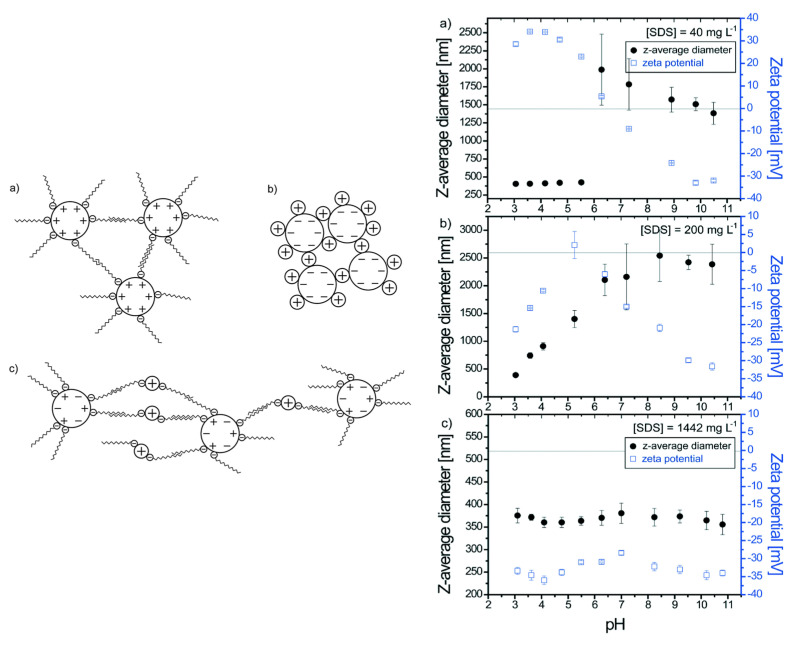
(**Left**) Schematic representations of TiO_2_ and SDS interactions and agglomerate formation. (**a**): TiO_2_–SDS agglomerates at pH 3.1. Hydrophobic interactions promote the formation of large agglomerates. (**b**): TiO_2_ agglomerate formation at pH 8.2 in the presence of divalent cations (⊕). Cation bridging between TiO_2_ promotes agglomeration. (**c**): TiO_2_–SDS agglomeration in the presence of divalent cations at pH 8.2. Cation bridging between SDS tails destabilizes the complexes. (**Right**) Z-average diameters and ζ-potential as a function of pH for (**a**): [SDS] = 40 mg L^−1^: charge neutralization and inversion is observed. SDS–TiO_2_ complex properties are mainly controlled by the TiO_2_ surface properties. (**b**): [SDS] = 200 mg L^−1^: the impact of SDS properties on the behavior of the TiO_2_–SDS complexes is more pronounced. Charge neutralization occurs and the isoelectric point is obtained at pH 5.2; by further increasing the pH, negative values are obtained, due to surface deprotonation. (**c**): [SDS] = 1442 mg L^−1^: the SDS–TiO_2_ complexes exhibit stable Z-average diameter and ζ-potential in the full pH range. [TiO_2_] = 50 mg L^−1^ (Reprinted from ref. [52]. Copyright 2017 The Royal Society of Chemistry).

In the case of titanium dioxide/distilled water nanofluids, the more stable dispersions were obtained when PVP was utilized as a stabilizer, whereas the use of the non-ionic surfactant polyoxyethylenesorbitan monolaurate (Tween 20) led to systems with lower viscosity; heat transfer is improved by both additives [51]. SDS also significantly influences the stability of TiO_2_ nanoparticles, via different processes, which include surface adsorption and agglomeration (Figure 3).

These processes are reversible (desorption, disagglomeration) when the pH or the SDS concentration changes, whereas the concentration of the surfactants, the presence of divalent electrolytes, and the mixing procedure (successive or punctual addition) are of significant importance, because of the complex interplay among the adsorption/desorption of the surfactant, specific adsorption, hydrophobic effects, charge cation bridging, inversion, agglomeration, and disagglomeration [52].

The anionic surfactant SDS was found to be the best among non-ionic (TritonX 100, PEG), anionic (SDS), and cationic surfactants (CTAB) in stabilizing ZnO in aqueous media, as its utilization resulted in particles with a smaller size distribution and longer resistance to sedimentation, especially following sonication [53]. In contrast, the non-ionic surfactant PVP resulted in smaller hydrodynamic radii of zirgonium oxide, ZrO_2_, compared to the ionic SDBS and to CTAB. PVP was found to create stable aqueous dispersions over a long period of time, with its concentration not playing a significant role [54]. Different concentrations of TiO_2_ were better dispersed when FBS was used as the surfactant in the conventional F-12K plus FBS cell culture medium, in comparison with cases where the non-ionic block copolymer pluronic F68 or the semi-synthetic plant-derived DPPC were used as anti-agglomerating agents [17]. In all cases, the size of the particles increased as a function of their concentration. Similar results were observed when nickel oxide (NiO) nanoparticles were investigated in the same media. Covalently bound dextran on the surface of permanently magnetic nanoplatelets ensured robust steric stabilization in different physiological buffers and in complex biological media. These kinds of nanoparticles are keen to agglomerate, not only because of the van der Waals attraction, but due to dipole–dipole interactions as well [34]. The presence of humic acid (HA) as the natural organic matter in conjunction with ultra-sonication (and, more specifically, the addition of the dispersant before the sonication) were critical for achieving a stable dispersion of TiO_2_ nanoparticles, together with the concentration of HA and the pH. At the same time, the optimum values of these parameters depend on the anatase or rutile crystalline phases of the nanoparticles [55].

**Figure 4:**
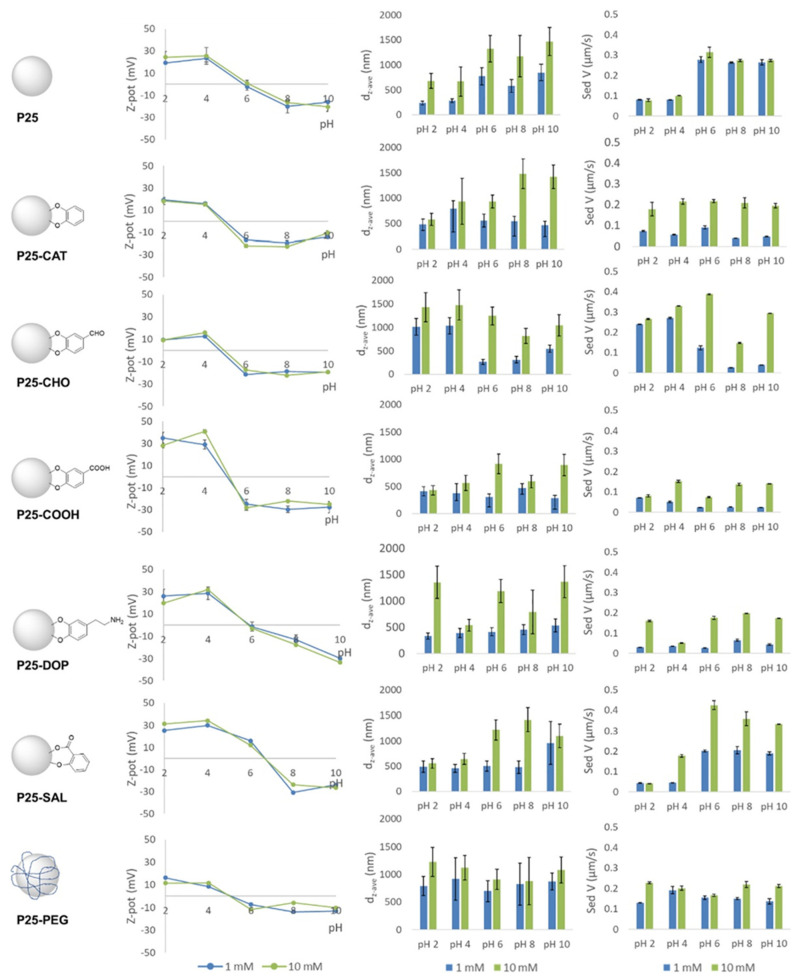
Zeta potential (Z-pot), hydrodynamic diameter (d_z-ave_), and sedimentation velocity (sed V) of pristine and functionalized Aeroxide^®^ P25 TiO_2_ nanoparticles (declared average particle size: 21 nm) dispersed in 1 and 10 mM NaCl solution for pH values from 2 to 10. Catechol (CAT), 3,4-dihydroxybenzaldehyde (CHO), 3,4-dihydroxybenzoic acid (COOH), dopaminehydrochloride (DOP), salicylic acid (SAL), and polyethylene glycol (PEG, M_v_ 100,000) were utilized for the functionalization. (Reprinted with permission from ref. [57]. Copyright 2018 Elsevier).

Magnetic iron oxide nanoparticles were also functionalized by the acidic form of sophorolipids [56]. No stable dispersions were achieved in the absence of sophorolipids, whereas when sophorolipids were employed, a stable colloidal suspension of maghemite Fe_2_O_3_ nanoparticles, in coexistence with a black/brown precipitate, was obtained; the presence of the precipitate was attributed to the nanoparticle aggregation before the addition of the sophorolipids and/or the insufficient complexation by the sophorolipids. An increase in temperature further assisted the dispersion. Different organic ligands have been utilized to influence the colloidal stability of TiO_2_ nanoparticles as a function of pH, electrolyte concentration, and dispersing medium, where different behaviors were observed depending on their functional group (Figure 4). It was shown that, in certain cases, the behavior was more influenced by the electrolyte concentration than by the pH, in contrast to other cases where, not only was the pH the main parameter, but it showed opposite effects for different modifiers. There were cases where none of these parameters were found to significantly influence the behavior or the final hydrodynamic radii measured in the dispersions [57].

Various mineral and complex test environments were used to examine the dispersibility of Ag nanoparticles [32]. In all liquid media, coated silver nanoparticles were significantly more stable compared to the uncoated ones. This was in agreement with the results of an independent study [58], which showed that uncoated Ag nanoparticles tend to precipitate in high ionic strength suspensions and sediment within a few hours. Furthermore, the dispersibility of both bare and surface-coated Ag nanoparticles with either poly(vinyl pyrrolidone) (PVP) or oleic acid (OA) was investigated, as well as its relation to bioaccumulation and reproductive toxicity in earthworms versus that of Ag ions [59]. Nanoparticles coated with PVP are hydrophilic and they usually form stable suspensions in polar solvents [60], whereas ones coated with OA are amphiphilic and form stable suspensions in both polar and non-polar solvents, as well as in polar/non-polar interface layers, depending on the pH of the suspension [61,62]. The primary particle diameters were determined by TEM, which showed that the OA-coated particles had a slightly smaller mean diameter than the PVP-coated ones. Dynamic light scattering measurements in DI water were in agreement with TEM concerning the size distributions of the PVP-coated nanoparticles, whereas they showed a greater ratio of larger aggregates for the OA-coated ones.

Surfactants also improve the stability of carbon nanomaterials (CNMs) in water, because of their adsorption via hydrophobic and π–π interactions. Ionic surfactants lead to stabilization of CNMs dispersions via the electrostatic repulsion between the charged hydrophilic head groups; a similar dispersion ability is obtained for both anionic and cationic types. Additionally, the purification process, as well as the surface-functionalization that defines the nanomaterial surface charge, influence the mechanism by which ionic surfactants can adsorb on the CNM surface. The phase behavior of carbon nanotubes (CNTs) in suspension depends strongly on the kind of surfactant used, its concentration, and on the type of interaction. Understanding the adsorption mechanism of ionic surfactants and the prediction of the colloidal stability of CNTs in different media requires knowledge of their surface charge. CNTs can be dispersed in water when coated by surfactants adsorbed on their surfaces, preferentially with those that have a relatively high hydrophilic–lipophilic balance [63]. The stability of aqueous dispersions of CNTs usually increases when sodium dodecyl sulfate (SDS) is utilized [64]. UV–vis spectroscopy has shown that the CNT/SDS dispersions exhibit very high stability; the amount of nanotubes in the supernatant liquid above the sediment decreased by only 15%, whereas the corresponding decrease in the case of bare CNTs was ~50% after 500 h was allowed for sedimentation. The interaction between CNTs and SDS via the hydrophobic segment results in a higher negative surface charge and steric repulsion, which enhances the stability of the CNT/SDS dispersion. It was, thus, concluded that a surfactant comprising of a single, long, straight-chain hydrophobic segment and a terminal hydrophilic group can be a suitable dispersant for stable CNT dispersions. Moreover, Tween 80 (T80), which is a non-ionic surfactant, was found to enhance the dispersion of multi-walled CNTs in aqueous media, whereas the presence of biological media, such as RPMI and DMEM cell culture media, improved the dispersion even further [65]. In that case, the stabilization was ascribed to steric effects, as there was no change in the zeta potential measurements.

#### 2.1.4. Dispersion Medium and Dispersibility

The effect of ionic strength (IS) and solution pH on nanoparticle dispersion has also been extensively studied, for example for anatase TiO_2_ nanoparticles with a primary particle size of 15 nm; the authors studied their influence on the hydrodynamic size and on the surface charge of the resulting ‘particles’ [66]. In one case, the nanoparticles were dispersed in NaCl solution with different concentrations to investigate the effect of the IS at constant pH and, in another, in solutions with the same ionic strength, but different pH adjusted by using HCl, NaOH and NaCl, and their combination. A large increase in the average size was found with increasing solution IS, since, at low IS, the electrostatic repulsive forces are dominant, whereas, when IS increases, the attractive forces dominate, resulting in a highly-agglomerated dispersion. Measurements of the average diameter of the TiO_2_ dispersions and of the zeta potential as a function of pH at constant ionic strength were also performed. For pH values far from the isoelectric point (IEP), a high value of zeta potential was measured, and the electrostatic repulsion prevailed over the van der Waals attraction and agglomeration was suppressed. For pH approaching the IEP, the low surface charge leads to a reduction of the repulsive forces, which results in an increase of the hydrodynamic size and in the formation of large flocs that sediment due to gravitational forces in a short time. Analogous conclusions were obtained when the aggregation of TiO_2_ was investigated for different concentrations of Suwannee river fulvic acid (SRFA) and various values of pH and ionic strengths [67]. The aggregation of bare TiO_2_ nanoparticles increased for pHs close to the zero point of charge, whereas at constant pH, aggregation increased with ionic strength. Furthermore, adsorption of SRFA resulted in a smaller degree of aggregation of the TiO_2_ nanoparticles, presumably due to enhanced steric repulsion. Dynamic light scattering showed that the TiO_2_ particles readily form stable aggregates at pH ~4.5 in a NaCl solution adjusted to an ionic strength of 0.0045 M [68]. At the same pH, when the ionic strength increased to 0.0165 M, micron-sized aggregates were formed within 15 min. At all other pH values, micron-sized aggregates were found to form faster than the minimum detection time of 5 min, even at low ionic strengths when NaCl was used. However, micron-sized aggregates form much faster in an aqueous suspension in the presence of CaCl_2_ than in respective suspensions in NaCl, showing that divalent cations may enhance the aggregation of titania.

Similar observations were made when the agglomeration of SiO_2_ nanoparticles in aqueous media was studied for different ionic strengths and pH values [69]. Addition of different salts (NaCl, MgCl_2_, BaCl_2_ and CaCl_2_) caused aggregation of the SiO_2_ nanoparticles, whereas a change of the pH within the range investigated did not influence the degree of aggregation in the absence of an electrolyte. The type of cation significantly affected the aggregation, with divalent cations (Mg^2+^, Ba^2+^ and Ca^2+^) being more efficient in destabilizing the nanoparticle suspension than the monovalent Na^+^ cations.

The effect of natural organic matter (NOM) on the aggregation of anatase TiO_2_ nanoparticles was also evaluated [70]. Changes in the particle size were measured as a function of the concentration of three different electrolytes (NaCl, Na_2_SO_4_, and CaCl_2_) and of the suspension pH. In general, the influence of the addition of an electrolyte in the absence of NOM followed DLVO theory. When the level of NOM adsorption on the titania surface was low, aggregation was induced, whereas an increase of the surface coverage could reduce the particle aggregation, even at high ionic strengths. The surface coverage was determined by the ratio of the concentration of NOM to that of the nanoparticles, whereas the mixing procedure was proven to be important, since it led to different final aggregation states. Ionic strength strongly influenced the aggregation behavior, whereas divalent cations and anions led to stronger destabilization of negatively or positively charged titania particles, respectively. Nanoparticles that were positively charged at low pH were more easily destabilized by SO_4_^2−^ compared to Cl^−^, whereas the opposite was observed for Ca^2+^ compared to Na^+^ for negatively charged nanoparticles at high pH. The addition of NOM at concentrations that create stable dispersions increased the stability of the suspensions with respect to Na_2_SO_4_ and NaCl but did not have much influence when CaCl_2_ was used.

The effect of concentration of sodium dodecylbenzene sulfonate (SDBS) surfactant and of pH on the size of ‘nanoparticles’ of alumina (Al_2_O_3_) and copper in water was investigated [71]. Optimal values of SDBS concentration (0.10% for alumina and 0.07% for copper) and pH (pH ~8.0 for alumina and pH ~9.5 for copper) were found, at which the effective particle diameters exhibited minimum values. Hexadecyl trimethyl ammonium bromide assisted in obtaining Cu nanoparticles with more than one order of magnitude smaller sizes in aqueous suspensions [72].

The degree of aggregation of CNMs increases at low pH, mainly due to the relatively smaller negative charge, although the degree of dispersion generally depends on the dispersing agent [35]. The dispersion of CNMs is significantly influenced by the presence of dissolved ions in water as well, where the aggregation of CNMs increases as the ionic strength increases, as expected. However, beyond a certain value of the ionic strength, there is no additional increase in the degree of aggregation, signifying that the electrostatic repulsive forces are already shielded. Moreover, increasing temperature results in an increase of the stability of CNM suspensions, most probably because of the disruption of weak interaction forces, increased Brownian motion (and, thus, collisions), and reduced zeta potential. Cellulose nanocrystals suspended in water also show pH-dependent size and viscosity; both quantities increase in acidic or alkaline conditions, whereas they obtain their lowest values at neutral pH [73].

Synthesized core-shell ZnS-coated CdSe nanocrystal quantum dots (QDs) were further coated to possess single -NH_2_, -COOH, -OH, or dual -NH_2_/OH and -OH/COOH functional groups [74]. The surface charge, as measured by zeta-potential measurements, varied depending on the functional group; it was found that QD-COOH and QD-OH/COOH were highly negatively charged, whereas QD-NH_2_ and QD-NH_2_/OH were positively charged. QD with hydroxyl groups were less negatively charged than the QDs with carboxylic acid groups, whereas QDs with both -OH and -COOH or -NH_2_ groups had median charge. QD-NH_2_ showed a broad particle distribution in contrast to QDs with -COOH groups that exhibited a much narrower distribution, while functionalization of the QD surface with -OH groups led to improved dispersion and stability under hypertonic conditions. In contrast, all QDs were stable in nonelectrolyte solutions. Moreover, all functionalized QDs were stable under weak alkaline conditions, whereas only QD-NH_2_ was stable under acidic conditions.

In conclusion, the investigation of the dispersibility of nanoparticles is a complicated process, since nanomaterials constitute dynamic entities that undergo physical and chemical transformations when mixed with environmental, synthetic, or biological media of different complexities, the characteristics of which affect the behavior to a large extend.

### 2.2. Solubility and Dissolution of Nanoparticles

The possibility of nanoparticles dissolving within the suspending medium is a key property that influences their toxicity and, consequently, their biological response, because it defines the fate of nanoparticles in the human body, as well as in the surrounding environment [75,76,77,78]. The solubility/dissolution of nanomaterials is frequently confused with their dispersion ability. Dissolution is defined as the dynamic process during which a particle dissolves in the matrix medium, in order to form a homogeneous solution or mixture [79]; this occurs when the constituent atoms or molecules have a specific solubility in the local environment. During this process, molecules from the surface of the dissolving nanomaterial are transferred to the solution forming a diffusion layer, which is the volume between the bulk solution and the solid nanomaterial surface that involves solvated molecules. The nanoparticle dissolution depends on the size [80,81] and the surface area [82,83], the surface morphology [77], the surface energy [84], the possible adsorbed species and the state of aggregation of the nanoparticles [85], as well as on the properties of the diffusion layer and the possible solute concentration in the suspending medium [79]. Furthermore, the dissolution kinetics depend on the size and, thus, the surface area as well, explaining why the dissolution of nanoparticles is faster and more extended in comparison with macroscopic particles of the same material [86,87].

**Figure 5:**
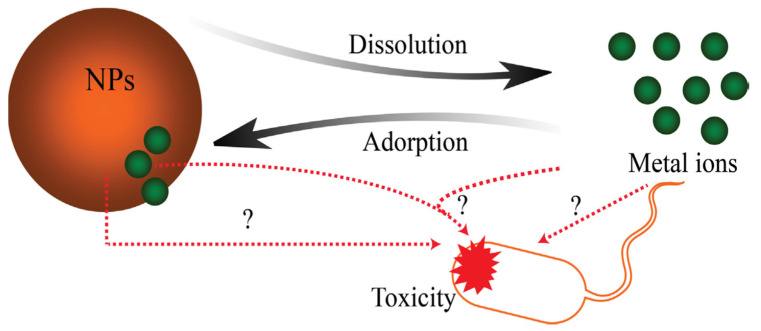
Nanoparticle toxicity can be attributed to the nanoparticles themselves, to released ions from the nanoparticles, or the combination of both. The procedures of dissolution and adsorption are both considered to contribute to the nanoparticle toxicity (Reprinted with permission from ref. [88]. Copyright 2016 Elsevier).

Nanoparticle antibacterial properties [88], toxicity [89], biomedical characteristics, and environmental impact [90] are strongly associated with their dissolution, since highly-toxic ions such as Zn^2+^, Cu^2+^, Cd^2+^, Ag^+^, etc. may be delivered to the solution [91,92,93,94]. It is possible, however, that a complex suspension—involving partially dissolved nanoparticles, free ions dissolved from the nanoparticles, and adsorbed ions on the nanoparticle surface—may be produced through the dissolution process in the surrounding media [95,96]. Figure 5 schematically illustrates that the metal oxide nanomaterial toxicity may originate from [88] the nanoparticles themselves, the released ions, or the combination of both, while adsorption of metal ions on the nanoparticles also affects toxicity. Moreover, since the nanoparticle surface interacts directly with biological systems, surface area is a key parameter of their biological effect [97].

**Figure 6:**
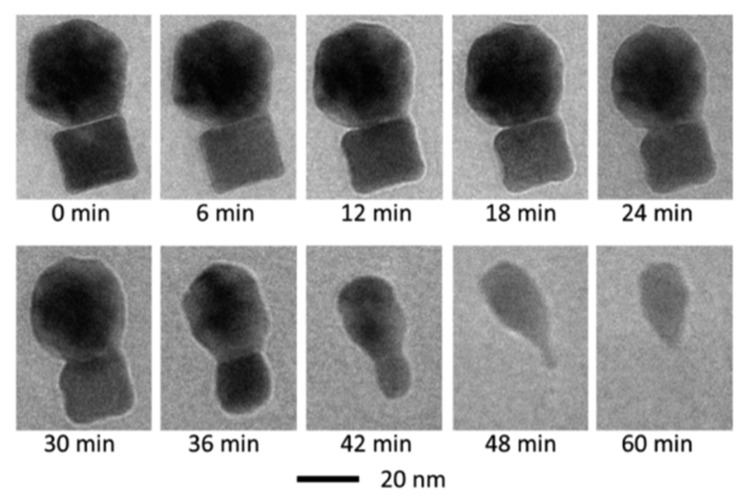
Morphological changes of icosahedral and cubic Pt Nanoparticles due to dissolution in the presence of aqueous solutions with a mixture of HAuCl_4_ and KCl. (Reprinted with permission from ref. [108]. Copyright 2017 American Chemical Society).

Generally, the dissolution of nanoparticles increases as the particle size decreases [98,99,100,101]. ZnO nanoparticles, however, do not exhibit major differences in their dissolution characteristics when compared to particles of micron size [102]; both nanoparticles and microparticles showed an 80% dissolution when added in Osterhout’s medium. It has also been reported in the literature that decreasing the particle size can reduce the extent of, or even prohibit, dissolution; when the dissolution of hydroxyapatite nanoparticles was studied as a function of particle size, it was observed that it was the larger particles that were prone to dissolution [103]. The dissolution of silver (Ag) nanoparticles, which affects their antibacterial properties, depends on their size. The smaller the Ag nanoparticles, the higher the dissolution rate, provided that aggregation of the nanoparticles is avoided, since this may lead to sedimentation. The formation of a passivation layer (e.g., an oxide layer) can inhibit their dissolution and, thus, their antibacterial activity [104]. The effects of the concentration and size of nanomaterials on the release of silver ions from citrate-capped Ag nanoparticles in a common hydroponic nutrient medium (quarter-strength Hoagland medium) was investigated, and the kinetics of ion release was accounted for by a kinetic model within hard sphere collision theory using the Arrhenius equation; thus, providing insight into the mechanisms of the ion release kinetics from the Ag nanoparticles [105]. Moreover, when the dissolution in water of PVP-stabilized and citrate-stabilized Ag nanoparticles was investigated [106], it was observed that the concentration of released silver ions was limited, whereas the dissolution rate and degree depended on the functionalization of the particles and on storage temperature. The dissolution is not only affected by the nanoparticle size, but by their shape and surface morphology as well [107]; when different shapes of CuO nanoparticles (spherical and rod shaped) were investigated, it was found that spherical nanoparticles dissolved faster and to a greater extent compared to rod shaped particles. The kinetics of dissolution due to oxidative etching of Pt nanoparticles of cubic and icosahedral shapes in aqueous solutions was investigated using a mixture of HAuCl_4_ and KCl as oxidative agent. Figure 6 shows the morphological changes of the icosahedral and the cubic Pt nanoparticles over a period of one hour. The shape of the nanoparticles was dramatically changed as dissolution proceeded. The corners became round and, after 1 h, the cube dissolved completely, while a small part of the icosahedron remained [108].

Nanoparticle dissolution is also affected by the parameters of the surrounding media, including pH, water hardness, ionic strength, temperature, and the presence of detergents or organic compounds [7,109]. For example, complete dissolution of CuO nanoparticles was observed in the presence of media enriched in amino acids [110], whereas cysteine was found to increase the Ag nanoparticle dissolution [111]. The solubility of copper-based nanoparticles was enhanced at low pH [112], whereas it was observed that ZnS nanoparticles showed the highest solubility at lower pH (in the range 9–10) and for the smallest particle size [113]. Moreover, at pH 7 (in DMEM), ZnO nanoparticles dissolved significantly more after 48 and 72 h when compared to suspensions at pH 4 (in Milli-Q water). When the ZnO nanoparticle accumulation inside A-431 cells was investigated, the authors presented arguments that the toxicity could be attributed to the nanometric size until 24 h of exposure, whereas, after 24 h (up to the 72 h of exposure was studied), both released Zn^2+^ ions and nanoparticles played an important role in the toxicity [83].

The dissolution of nanoparticles is strongly related with their bioavailability, degree of uptake, and toxicity [114]. The toxicity of nanoparticles is related to their chemical characteristics and surface chemistry [115,116]; this is due to the possibility of releasing toxic ions and/or the production of reactive oxygen species (ROS) [117]. Toxic effects through the production of ROS are very likely to occur for nanoparticles of small size and, thus, of large reactive area. Nevertheless, when the dissolution of nanoparticles takes place during the cell culture, it is difficult to identify the origin of the toxic effects. The toxicity of a number of particles was tested in relation to their dissolution. The authors categorized the nanoparticles into soluble (Ca_3_(PO_4_)_2_, Fe_2_O_3_, ZnO) and insoluble (CeO_2_, TiO_2_, ZrO_2_), and studied the cytotoxicity on two different cells lines; it was found that, for high dissolution, the toxic effects were considerably higher compared to those for little or no dissolution [118].

The solubility of ZnO nanoparticles, with an emphasis on the toxicological effects of zinc ions, has been widely studied [119]. It has been reported that the higher the nanoparticle dose, the more the cell nuclei are condensed, leading to cell apoptosis [120]. ROS, such as hydrogen peroxide, superoxide anions, hydroxyl radicals, and organic hydroperoxides, can be produced in an aqueous suspension of ZnO nanoparticles; these ROS can cause injury to cells, whereas they also display a strong antibacterial activity [100]. Cytotoxicity studies of ZnO, CeO_2_, and TiO_2_ nanomaterials and their relation to dissolution suggested that the toxicity induced by ZnO nanoparticles is due to the dissolution of the ZnO nanoparticles in the aqueous environment and the release of Zn^+^ in the culture medium, which is associated with high levels of ROS. On the other hand, CeO_2_ showed a cytoprotective behavior by suppressing ROS production; this led to cellular resistance to the oxidative stress. Finally, TiO_2_ was considered inert, since it did not result in toxic effects on mammalian cells [121]. To evaluate the toxicity in marine diatoms, ZnO nanoparticle dissolution has been examined in seawater; the toxicity was attributed to the ZnO dissolution that released zinc cations [122]. Even inert nanoparticles can induce ROS under living conditions; this is due to their ability to target mitochondria. A number of cellular events can be influenced by ROS, such as signal transduction, proliferation rate, gene expression, and protein redox regulation. At high ROS levels, cells may be damaged by altering proteins, deoxidizing lipids, or disrupting DNA, which can even lead to cancer due to gene transcription modulation [120,123]. The dissolution of ZnO nanoparticles, their uptake, and the routes they follow to enter LoVo cells has also been investigated. It was found that ZnO nanoparticles can enter LoVo cells by passive diffusion, endocytosis, or both, according to their agglomeration state. When ZnO nanoparticles contact the acidic pH of the lysosomes inside the cells, zinc ions are released. These ions together with the presence of ZnO nanoparticles produce ROS that cause DNA damages. Thus, the ZnO nanoparticle toxicity is attributed to a combination of the presence of the particles and of the zinc ions [124]. ZnO nanoparticle dissolution has been studied in various biologically relevant solutions, including HEPES, MOPS, and PIPES, where it was discovered that the buffers affect the dissolution kinetics and toxicity of the nanoparticles. Experiments on cell viability have shown that the use of buffers decreases the viability of Jurkat leukemic cells after the introduction of ZnO nanoparticles [125].

The dissolution of silver nanoparticles starts immediately upon exposure to the particular medium and continues for several hours. The oxidative dissolution of Ag is also responsible for the toxicity of the nanoparticles, which is ion- and particle-related [77]. The oxygen present induces the formation of Ag_2_O on the surface of the silver nanoparticles and the release of silver cations in the aqueous solution. Moreover, low pH and smaller particle size enhance the Ag nanoparticle dissolution [126]. In general, different forms of silver may be contained within a suspension of Ag nanoparticles, such as free or complexed Ag^+^ and Ag^+^ adsorbed on the nanoparticles. The state of Ag nanoparticles in pure water or an aqueous nitric acid environment was investigated for a range of pHs, between 0.5 and 6.5 [127]; the findings suggest that the dissolution of silver nanoparticles depends on the particle size, since larger particles did not dissolve in nitric acid for concentrations up to 4 M, whereas faster reaction rates occurred with increasing temperature. The effect of chlorine anions on Ag nanoparticle dissolution, generation of ROS, and toxicity of Ag nanoparticles has also been investigated, since chlorine anions are the most common anions in aqueous systems. It was found that high concentrations of chlorine anions facilitate the dissolution and toxicity of the nanoparticles, because of the formation of Ag−Cl complexes [117]. Ag nanoparticle toxicity has also been examined for both positive and negative surface charges. The results revealed that the nanoparticles with positive charge were less toxic to tumor cell lines, even though they exhibited ion release rates similar to those of negatively charged nanoparticles. However, the cytotoxicity of Ag nanoparticles is a combination of events, which include, apart from the surface charge, the release of silver ions, the dissolution rate, and the activity of biological molecules [128].

Nanoparticles that dissolve in the medium before their uptake by organisms may have ion channels as a route for cellular entry [96]. The nanoparticles that resist complete dissolution follow other routes to influence the fate of cells, such as endocytosis, ion transportation, or both. Nanoparticle dissolution may also occur after cell uptake and inside the cells (intracellular dissolution); this is strongly dependent on the nanoparticle shape [129]. This dissolution mechanism shows how nanoparticles bypass the good protection of mammalian cells, as well as how heavy metal ions conduct themselves inside cells. Copper metal nanoparticles stabilized using a carbon layer were tested for the effects of nanoparticle dissolution on cytotoxicity and were compared to the behavior of copper oxide nanoparticles. The influence of pH on the solubility was studied using artificial buffer solutions of pH 5.5 and pH 7.4. At neutral pH, almost no free copper ions could be detected after 3 days in the cell culture medium, confirming the stability of the particles. However, at an acidic pH of 5.5, as found inside lysosomes, the copper oxide particles dissolved rapidly, whereas the fairly stable carbon-coated copper particles released copper to the surrounding medium. Thus, intracellular dissolution was attributed to pH effects [99].

Dissolution of nanoparticles is one of the main contributors to particle toxicity. The dissolution process may occur inside or outside cells. Nanoparticles dissolve mainly by releasing ions, which are possibly toxic for living organisms. Nanoparticle dissolution can be affected by the chemistry, size, shape, and surface coating of nanoparticles, as well as the type of media, the pH, and the solution characteristics of the surrounding environment.

### 2.3. Hydrophilicity–Hydrophobicity

The hydrophilic/hydrophobic behavior of nanomaterials is mainly associated with their chemical features, such as composition and surface charge, as well as their surface coating characteristics, stability, and surface reactivity. The wetting characteristics of nanoparticles are critical for their biological application [130,131] and are often strongly related to their biocompatibility and their dispersion and interaction with biomolecules [132]. The hydrophobic interaction is generally thought to be the strongest among all long-range non-covalent interactions in all aqueous systems, as well as in biological ones. It is advantageous for the adsorption of biomolecules, promotes the interaction and adhesion with cell membranes by increasing nanoparticle uptake for cellular delivery, and adjusts the release rate of drugs [133,134].

The modification of the wetting characteristics of a nanoparticle surface can be realized during either the nanoparticle synthesis or by the post-preparation of surface coatings on the nanoparticles using appropriate polymers or surfactants. Synthetic procedures in the presence of block or graft copolymers with hydrophilic segments can lead to hydrophilic surface coatings; polymeric surfactants used include poly(ethylene glycol) (PEG), poloxamers, poloxamines, polysaccharides, and nonionic surfactants, such as polysorbate 80 (Tween 80) [130]. Alternatively, post-preparation coating of the nanoparticle surface using hydrophilic polymers or surfactants is commonly achieved through chemisorption or covalent attachment of polymers or surfactants with a functional end-group to a reactive surface (grafting-to) or by in situ polymerization of monomers from immobilized initiators onto the nanoparticle surface (grafting-from) [135]. Hydrophilic homopolymers and copolymers and other coupling agents are also used to affect, both the nanoparticle morphology, and its surface modification, as well as to introduce specific functional groups on the nanoparticle surface; these agents can be silane coupling agents, titanate coupling agents, and organophosphonic acids [136,137].

**Figure 7:**
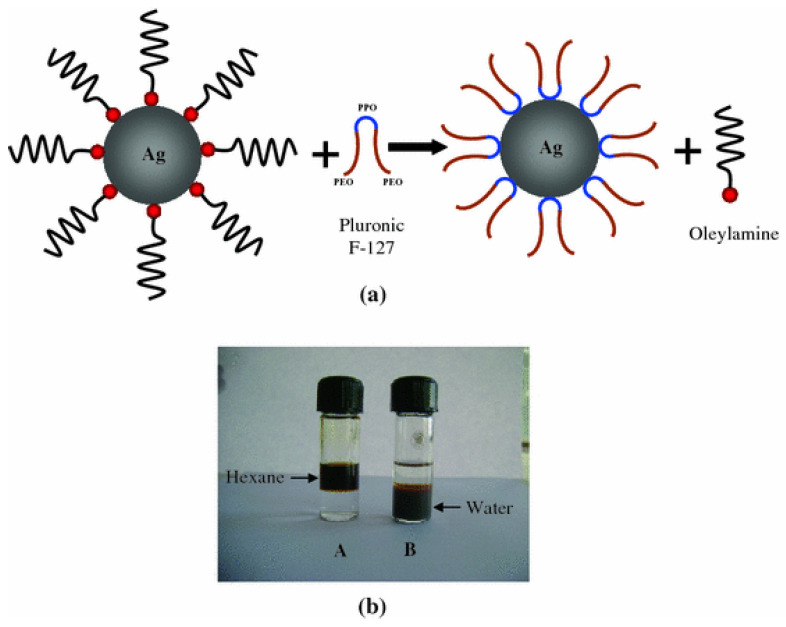
(**a**) The process of modifying the wetting characteristics of Ag nanoparticles from hydrophobic to hydrophilic using pluronic F-127 surfactant. (**b**) Ag nanoparticles before and after the phase transfer (Reprinted with permission from ref. [131]. Copyright 2010 Springer).

Among all known nanomaterials, silver nanoparticles exhibit the highest biocompatibility and antimicrobial activity. One synthetic method utilizes the thermal reduction of AgNO_3_ in the presence of oleylamine as a reducing and capping agent [131]; the adsorption of oleylamine on the surface of the nanoparticles makes them hydrophobic, as illustrated in Figure 7. In order to increase the dispersibility of such hydrophobic nanoparticles in water, a facile phase transfer mechanism has been developed using pluronic F-127, a biocompatible block copolymer [131]. Modifying the Ag nanoparticles surface utilizing PVP allows the formation of suspensions stable in polar solvents [60], whereas using an amphiphilic surfactant, such as oleic acid, allows suspensions stable in polar solvents, in non-polar solvents, and in polar/non-polar interface layers [61,62].

Single and multi-walled carbon nanotubes (CNTs), with diameters between 0.4 and 2 nm, and 2 and 100 nm, respectively, could potentially be utilized in a wide range of biological and biomedical applications. One of the main technical obstacles for the use of CNTs in these fields is their extremely low dispersibility in aqueous solutions. A number of methods have been used to alter the surface of CNTs, in order to modify their wettability and introduce a hydrophilic character, with the most common being functionalization with hydrophilic polymers [138]. Oxidative acid treatment can introduce nanotube-bound carboxyl acids, thus, enabling defect-targeted functionalization. Esterification, amidation, ionic interaction treatments, and sidewall-targeted functionalization of CNTs are most commonly realized by surface-attaching hydrophilic polymeric or oligomeric species onto nanotubes. PEG, poly(vinyl alcohol) (PVA) and poly(propionylethylenimine-co-ethylenimine) (PPEI-EI) have been utilized to functionalize SWCNTs. The hydrophobicity of CNTs can also be modified using non-covalent or covalent modification with carbohydrates (monosaccharides and polysaccharides), proteins, and nucleic acids [139]. Short double-stranded DNAs and certain RNAs have been used to directly disperse individual SWCNTs in water [140], where the interactions of nucleic acid with the SWCNTs in the aqueous media originate from the stacking of the bases of the nucleic acids on the nanotube surface with the hydrophilic sugar-phosphate backbone pointing towards the solution, to achieve solubility in water. The use of sodium dodecyl sulfate (SDS) as a dispersing agent allows the preparation of hydrophilic CNTs. The hydrophobic hydrocarbon segment of SDS interacts with the CNTs, where the hydrophilic sulfate group causes a high negative surface charge and steric repulsion that improves the stability of the CNT/SDS dispersion [64]. Hydrophilic MWCNTs decorated with magnetic nanoparticles have also been prepared by first synthesizing poly(acrylic acid)-functionalized MWCNTs (PAA-g-MWCNTs) and then decorating these with magnetic nanoparticles, utilizing chemical co-precipitation of Fe^2+^ and Fe^3+^ onto the outer surfaces of the PAA-g-MWCNTs; they exhibited an exceptional dispersion ability in water, as well as high magnetic susceptibility [141].

Silica nanoparticles are well known for their hydrophilicity and biocompatibility. However, often it is necessary to make them very hydrophilic. Generally, the presence of silanol groups on the surface of SiO_2_ makes nanoparticles more hydrophilic and, consequently, more easily dispersible in aqueous media [142]. The addition of organosilane compounds containing PEG chains onto silica nanoparticles led to highly hydrophilic and more easily dispersible nanoparticles [143]. Alternatively, silica nanoparticles can be modified with other polymers soluble in water, such as poly(oxyethylene methacrylate) (POEM) and poly(styrene sulfonic acid) (PSSA) [135]. In this case, the process includes three steps: activation of the silanol surface groups of the SiO_2_ nanoparticles, surface alteration to chlorine (-Cl) groups, and grafting-from polymerization of the polymer chains. The nanoparticles after modification exhibited better dispersibility compared to the unmodified ones [135]. Furthermore, polystyrene-functionalized silica nanoparticles have been prepared via radical polymerization of styrene monomer onto nanoparticles possessing vinyl groups, with benzoyl peroxide as the initiator, resulting in PS-g-SiO_2_ particles. These PS-g-SiO_2_ nanoparticles were easily dispersed in organic solvents such as methylbenzene, whereas when deposited onto a silicon wafer, they resulted in a superhydrophobic surface [144]. Hydrophilic silica nanoparticle surfaces have also been turned hydrophobic with the addition of alumina sol. The degree of hydrophilicity of the produced silica-alumina nanoparticles was controlled by changing the proportion of alumina. It was shown that the nanoparticles modified with 1, 2, and 5% alumina gained 5, 2, and 1% weight in water compared to the unmodified particles, where the gain was 8% [145].

**Figure 8:**
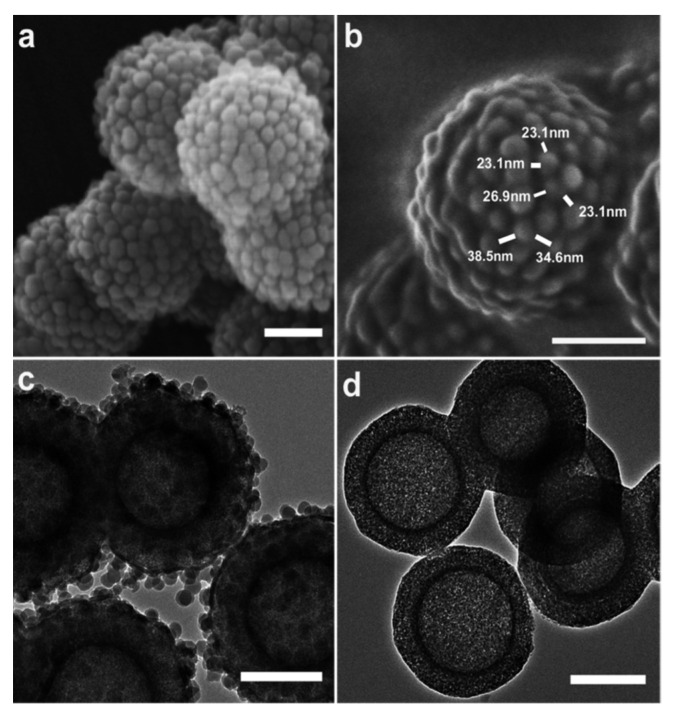
Morphology of the surface of RHMS and MHS nanoparticles. (**a**) SEM image of RMHS, (**b**) high-resolution SEM (HRSEM) image of RMHS, illustrating the distances between neighboring shell silica nanospheres, (**c**,**d**) HRTEM images of RMHS and MHS, respectively. Scale bar = 200 nm (Reprinted with permission from ref. [134]. Copyright 2015 American Chemical Society).

Production of nanoparticles with hydrophilic composition and hydrophobic properties at the nanoscale level has been attempted by employing surface topology engineering (Figure 8). This takes advantage of the fact that surface roughness affects the wettability behavior. Thus, mesoporous hollow silica (MHS) nanospheres with controlled surface roughness (rough mesoporous hollow silica, RMHS) have been produced by introducing silica shell particles with smaller sizes of O(10nm) onto MHS with relatively larger sizes of O(100 nm). These rough MHS nanoparticles exhibited an unexpected hydrophobicity in comparison with the respective MHS with no roughness, and this led to higher adsorption of a range of hydrophobic molecules and the sustained release of hydrophilic drugs [134].

Hydrophobic barium sulfate (BaSO_4_) nanoparticles were produced using a one step process that combined their synthesis and surface modification [137]. The nanoparticles were produced by a precipitation reaction of calcium chloride and ammonium sulfate in a aqueous solution using the modifying agent octadecyl dihydrogen phosphate (n-C_18_H_37_OPO_3_H_2_, ODP). The produced nanoparticles were hydrophobic because of the formation of a thin layer of barium alkyl phosphates on the nanoparticle surface during synthesis. It is noted that barium alkyl phosphates control the particle size and morphology of nanoparticles as well.

Iron oxide nanoparticles are of great importance in biomedical applications, such as bioimaging, drug delivery, cellular therapy, etc., due to the possibility of surface modification and their low toxicity [146,147]. With no surface coating, the surfaces of these nanoparticles are hydrophobic, and exhibit a large surface to volume ratio [148,149]. These particles tend to agglomerate because of hydrophobic interactions and form large clusters in aqueous media, which also significantly affects their magnetic properties. To overcome this, a variety of surface coatings have been employed to alter the nanoparticle surface, whereas, for effective stabilization, often a very high surface density for the coating is required. One approach, is to add some stabilizer, such as a surfactant or a polymer, at the time of preparation, to prevent aggregation of the nanoscale particulates. Alternatively, the particles can be modified after precipitation. Among the most common surface modifiers are synthetic (e.g., PEG, PVP, PAA, PVA, etc.) or natural polymers (e.g., dextran, chitosan and gelatin), fatty acids, polypeptides, and inorganic coatings [150].

When nanoparticles come into contact with biological fluids, they are coated with proteins within seconds; therefore, cells or tissues almost never interact with the bare particles [151,152]. The protein–nanoparticle interactions, which form the so-called nanoparticle–protein ‘corona’, have a key role in nanomedicine [153]. The proteins that are present in the plasma are adsorbed onto the nanoparticle surface, depending on the nanoparticle surface characteristics; this is crucial for their in vivo distribution [154]. The hydrophobicity of the nanoparticles affects both the quantity and the composition of the plasma protein adsorbed layer. Nanoparticles with decreasing surface hydrophobicity were studied with respect to their influence on plasma protein adsorption [155]. Latex particles with different hydrophobicities were used as model colloidal carriers; it was found that, when the surface hydrophobicity decreased, the quantity of adsorbed proteins decreased and the changes in the obtained protein adsorption patterns deteriorated. The hydrophobicity of copolymer nanoparticles (70–700 nm) was controlled via the co-monomer ratio of N-isopropyl-acrylamide and N-tert-butyl acrylamide (NIPAM/BAM) in the copolymer synthesis, where the NIPAM-rich particle was the most hydrophilic, and the adsorption of human serum albumin (HSA) onto these nanoparticles was investigated. The more hydrophobic nanoparticles were completely covered with a single layer of HAS, whereas particles with 25% BAM or less exhibited very little binding of HSA [150].

In the fields of nanomedicine and therapeutics, the successful cell uptake of nanoparticles and, consequently, the interaction of nanoparticles with the cell membrane is vital. The wetting characteristics of nanoparticles play a key role in cell uptake, since their interaction with the cell membrane depends not only on their shape, surface chemistry, and geometry but also on their hydrophobicity [156,157]. Small molecule nanoparticles (SMNPs), prepared by self-assembly of π-conjugated oligomers with varying degrees of hydrophobicity, were electroporated into live HeLa cells. It was observed that the more-hydrophilic SMNPs disassembled and dispersed upon cellular uptake cell, whereas the hydrophobic ones remained intact within the cells [158]. It has been shown that the bioactivity of synthetic nanoparticles can be improved with the introduction of hydrophilic co-monomers in the hydrophobic segment; the introduction of 2-hydroxyethyl methacrylate in the hydrophobic block of a poly(ethylene glycol)-block-poly(α-tocopheryl succinate) reduces the hydrophobicity of the corresponding nanoparticles and increases their bioactivity [159]. TiO_2_ nanoparticles, which are used in oral applications, were tested for their wetting behavior in relation to their cell–nanoparticle interactions. The viability of epithelial cells, when in contact with either hydrophobic or hydrophilic nanoparticles, was not affected. However, the hydrophobic nanoparticles aligned to the cell membrane, wrapped up and were found in endosomes and lysosomes, while the hydrophilic nanoparticles directly entered the cells and were found in the cytoplasm [160].

## 3. How the Key Parameters Affect Functionalities with Respect to Applications

### 3.1. Cellular Uptake

#### 3.1.1. Mechanisms of Cellular Uptake

Nanomaterials that circulate in a multicellular living organism interact with its components in a fundamentally different way compared to the soluble small molecules or micron-scale particles that are recognized by the immune system [161,162,163,164]. Materials at the nanoscale can interact with the endogenous cellular machinery through active energy-dependent processes that selectively move substances against their electrochemical gradient across cell membranes [165,166,167,168,169,170,171,172]. Endocytosis is the mechanism of actively transporting cargoes into the cell in transport vesicles derived from the plasma membrane [165]. The different mechanisms of endocytosis are generally classified as phagocytosis and pinocytosis. Phagocytosis is the predominant mechanism used mainly by macrophages and less frequently by nonprofessional phagocytes, including epithelial cells, fibroblasts, and endothelial cells [173]. Pinocytosis is present in all types of cells, in forms such as macropinocytosis, which enables the uptake of large NPs that seems impossible via other endocytosis pathways [174]; caveolae-dependent endocytosis; clathrin-dependent endocytosis; and clathrin- and caveolae-independent endocytosis, with the last three forms referred to as receptor-mediated endocytosis [175,176,177]. The phenomena taking place at this nano–bio interface result in the modulation of cell fate, the induction or prevention of mutations, the initiation of cell–cell communication, and the modulation of cell structure [178,179].

**Figure 9:**
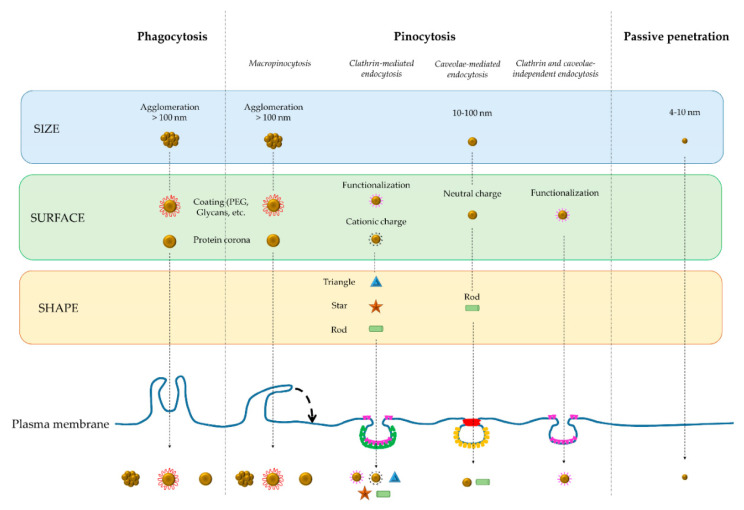
Different cellular internalization mechanisms in relation to the nanoparticle properties, such as size, surface functionality, and shape. The cell can internalize the nanoparticles by using different mechanisms, taking into account the same parameters [190].

It has been extensively reported in the literature that the uptake of nanoparticles by the cells depends on the nanoparticle characteristics, including the size and/or shape, the surface charge, and surface hydrophobicity [178,180]; on the possible sedimentation of large and dense particles, on the properties of the protein corona of the individual nanoparticles [161,162,166,181,182,183,184,185,186,187,188]; and, finally, on the cycle phase of the living cell [189]. The nanoparticle properties mainly designate their endocytosis route, but, in many cases, the cell can internalize the nanoparticles by utilizing distinct mechanisms, which are also related to these parameters, as illustrated in Figure 9 [190].

The effect of size on the cellular uptake of nanomaterials is a central issue in the field of Nanobiology [191]. In this context, for the development of suitable cell-tracking and drug-carrier nanoparticle systems, nanoparticle size is considered an important parameter, since it determines the mechanism and rate of cellular uptake of the nanoparticle and its ability to permeate through tissues [192,193]. An equation has been formulated to calculate the minimum radius of a nanoparticle (*R*_min_) required for full wrapping; this *R*_min_ is determined by the energy released from the ligand-receptor binding (adhesion strength) and the energy needed to bend the membrane (membrane rigidity). Thus, the dependence of cellular uptake on the nanoparticle size and shape has been extensively investigated [194].

#### 3.1.2. Effects of Geometrical Characteristics on Cellular Uptake

Well-dispersed amorphous silica nanoparticles were utilized to investigate their uptake, localization, and cytotoxic effects in mouse keratinocytes (HEL-30) [195]. In that study, the cells were cultured for 24 h using different concentrations of SiO_2_ nanoparticles with an 30–535 nm average particle size; the cells were assessed for particle uptake and biochemical changes. TEM analysis revealed that all silica particles were successfully taken up into the cells independently of size and were localized into the cytoplasm. Moreover, the interplay between silica nanoparticles of different sizes affecting the cellular uptake with Hela cells in serum-free medium has recently been reported [196]. When the cells were co-exposed to silica nanoparticles of different sizes, the bigger nanoparticles significantly promoted the cellular uptake of the smaller ones, while the smaller nanoparticles inhibited the cellular uptake of the larger ones. In fact, this was observed, even when the effects of size were very small or undetectable in the single-exposure experiments.

When surface-functionalized pomegranate-like ferrimagnetic nanoclusters (40–85 nm) were used in vitro, it was shown that the proliferation of spleenocytes, as well as the cytokine production, were consistent with the regulation of immune system cells based on size; it was inferred that small clusters mainly drive immune-stimulatory and inflammatory responses, while large ones could lead to immune-suppressive and anti-inflammatory actions [197].

The effects of the size and surface charge of polymeric nanoparticles on cellular uptake and biodistribution have been investigated [185]. Murine macrophages were found to more efficiently phagocytose nanoparticles with a large size and high surface charge. Even minor differences in the size and/or the surface charge of the nanoparticles had a significant impact on their cellular uptake activating different mechanisms in the endocytosis process. In vivo biodistribution indicated that 150-nm nanoparticles with small negative charge showed a tendency to accumulate more efficiently in tumors [185].

The cellular interactions of biologically-active gold nanoparticles as a function of size in the range of 15–55 nm with alveolar macrophages were evaluated. These cells, as professional phagocytes, are the first line of host defense in the lungs, and their potential role in initiating oxidative stress has also been studied. In vitro exposure resulted in morphologically unusual sizes and adherence characteristics, with significant uptake of nanoparticles at high doses after 24 h [198].

Significant differences were observed concerning the uptake of colloidal gold nanoparticles of different sizes and shapes [181]. More specifically, the intracellular concentrations of rod-shaped nanoparticles (74 × 14 nm) differed from those of either 74 or 14 nm spherical nanoparticles. These results were attributed to the difference in the curvature and the active surface area between rod-shaped and spherical nanoparticles: the rod-shaped nanoparticles actually have a larger contact area with the cell membrane receptors than the spherical ones when the longitudinal axis of the rods interacts with the receptors. An alternative explanation is related to differences in the distribution of the surfactant molecules adsorbed on surfaces with different curvatures during the synthesis of the nanoparticles, which may affect the homogeneity of the serum protein coating and, thus, the effective binding to receptors [181].

**Figure 10:**
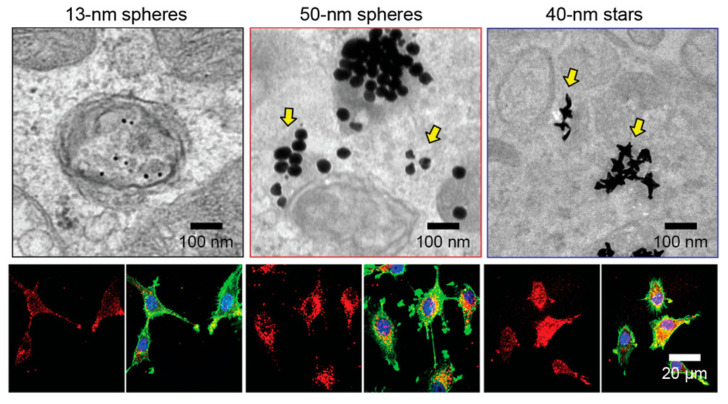
Dependence of the yield of cellular uptake and the intracellular distribution of gold nanoparticle–siRNA constructs on nanomaterial size and shape. In vitro response of U87 glioblastoma cells to various types of nanoconstructs. Transmission electron microscopy (TEM) images (**top row**) and confocal fluorescence microscopy images (**bottom row**) revealing the 13-nm spheres located within endocytic vesicles, with the 50-nm spheres and 40-nm stars being aggregated, and some being outside of the endocytic vesicles (yellow arrows in top row). In the fluorescence images, the actin cytoskeleton and the nucleus were stained with Alexa Fluor 594 Phalloidin (green) and DAPI (blue), respectively, whereas the nanoconstructs were labeled with Cy5 (red) (Reprinted with permission from Ref. [204]. Copyright 2017 American Chemical Society).

Generally, it is suggested that the receptor–ligand binding constants, the receptor recycling rates, and exocytosis can be mediated by the size and the shape of the nanoparticles. A significant number of studies have shown that geometry, in addition to the size of nanoparticles, determines the rate of uptake and, importantly, the uptake mechanism used by nanoparticles. More specifically, experimental studies using different cell types have shown that spherical nanoparticles undergo a higher cellular uptake than rod-shaped nanoparticles [181,182,199]. Moreover, some cylindrical nanoparticles of different materials, such as carbon nanotubes, iron oxide, and polymers, have demonstrated enhanced circulation and retention times compared to their spherical counterparts [200,201,202,203]. The in vitro responses of U87 glioblastoma cells to various types of gold nanomaterials (13-nm spheres, 50-nm spheres, and 40-nm stars) conjugated with siRNA were studied; a much higher uptake efficiency was observed for the 50-nm spheres and the 40-nm stars when compared to the 13-nm spheres, as illustrated in Figure 10 [204].

The geometry of nanoparticles appears to also affect the mechanism of their endocytosis. Cellular uptake inhibition experiments indicated that the endocytosis of spherical silica nanoparticles is mainly carried out by a clathrin-mediated mechanism, while most of their rod-like counterparts penetrate the cell membrane via macropinocytosis or phagocytosis [205]. However, functionalization of the nanoparticles seems to modify the manner of their internalization [206,207].

Saturation of the intracellular nanoparticle concentration within hours has been reported [181,208], whereas other reports indicated saturation after several days [209,210,211]. Moreover, the kinetics and the saturation concentrations were reported to strongly depend on the nanoparticle dimensions [181]; however, the saturation rate of their uptake seemed to depend on the number of available free proteins, which are not adsorbed on the nanoparticle surface in the medium, since these unbound proteins may compete for the receptor binding sites of the cell surface with those proteins adsorbed on the nanoparticle surface.

In order to avoid complications due to the sedimentation of nanoparticles in typical cell cultures, upright and inverted cell culture configurations were utilized. These kind of cell experiments illustrate that the cellular internalization of gold nanoparticles depends on their sedimentation and diffusion velocities and not on their size, shape, surface coating, density, and initial concentration. It was also found that more nanoparticles were endocytosed in the upright configuration than in the inverted one, whereas larger differences in uptake between the two configurations were observed for nanoparticles exhibiting faster sedimentation rates. It is, therefore, considered that for in vitro studies with large and/or heavy nanoparticles, sedimentation needs to be taken into serious consideration.

#### 3.1.3. Effects of Surface Charge and Surface Coating on Cellular Uptake

Experimental and theoretical studies have investigated the effect of charge, hydrophobicity, and interfacial forces on the interaction between nanoparticles and lipid bilayer assemblies, in order to understand the interactions of the nanoparticles with the membrane and the mechanisms that affect their cellular influx, as well as the cytotoxicity and inflammatory effects [180,212,213,214,215].

Molecular dynamics simulations confirmed that electrostatic interactions dominate over the hydrophobic ones when considering nanoparticles, with the bilayer with charged nanoparticles interacting more favorably than their uncharged counterparts. More specifically, the adhesion of anionic nanoparticles more strongly influences the membrane structure when compared to cationic nanoparticles, which can promote local disorder in the area of adhesion, as well as membrane-wrapping phenomena [216,217]. In another study, computed results indicated that the initial orientation of non-spherical nanoparticles can be significantly affected by surface charge density; thus, enhancement of the translocation rate and maximizing the cell adhesion can be achieved by engineering the interplay of nanoparticle shape and surface charge density [218].

**Figure 11:**
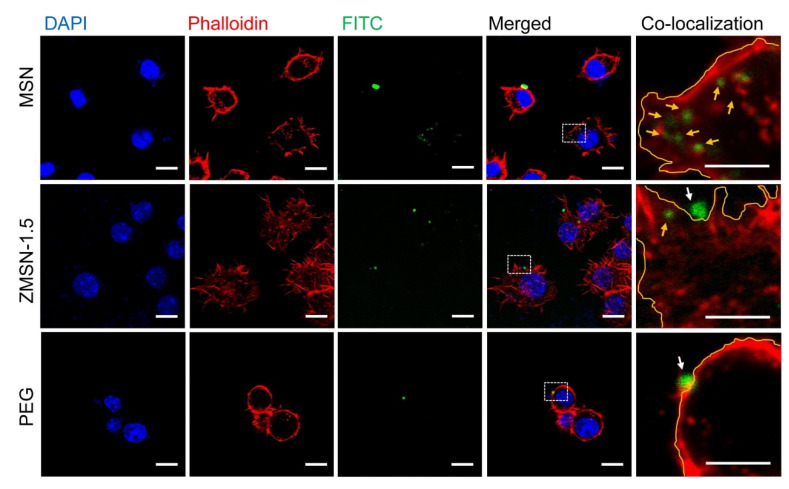
Dependence of the cellular uptake of bare mesoporous silica nanoparticles (MSNs), pseudo-zwitterionic ZMSN-1.5, and control PEGylated MSNs by RAW 264.7 macrophages. Laser scanning confocal microscopy images of the nuclei (DAPI), membrane (Phalloidin), and nanoparticle (FITC) emission channels are shown. Merged images and high magnification merged red-green channels overlain allow co-localizing the different systems studied. In the co-localization right row area, selection of region of interest was made with FiJi, marking in yellow the cell membrane border. Internalized nanoparticles are highlighted with yellow arrows, while those located in the outer area are marked with white ones (Scale bar: 10 μm, 5 μm for co-localization row) (Reprinted with permission from Ref. [222]. Copyright 2019 Elsevier).

Additionally, a number of experimental studies have elucidated the impact of surface charge on the interaction between nanoparticles and cell membranes. In agreement with theoretical models, it has been shown experimentally that cationic nanoparticles strongly bind to the cell membrane, through electrostatic interactions with the lipid phosphate groups, increasing the surface tension of the membrane and resulting in the formation of pores [219]. It has also been reported that negatively or positively charged nanoparticles preferentially interacting with the choline-phosphate dipole (N^+^/P^−^ terminus) of the lipid membranes, respectively, could cause the surface reconstruction of phospholipid membranes [220]. Charged nanoparticles tend to adsorb more proteins from the serum compared to neutral nanoparticles [180]. It was demonstrated that large amounts of plasma proteins were adsorbed on positively- or negatively-charged decorated gold nanoparticles, whereas relatively few proteins adsorbed onto neutral ones [221]. Mesoporous silica nanoparticles (MSNs), such MSNs modified with two different silanes, in order to produce mixed-charge amino-phosphonate pseudo-zwitterionic MSNs under physiological conditions (ZMSN-1.5) and of PEGylated MSNs were studied with respect to their internalization by flow cytometry and laser scanning confocal microscopy experiments. It was shown that cell uptake was drastically reduced for the functionalized nanoparticles, both for the pseudo-zwitterionic ZMSN-1.5 and for the PEGylated ones; this is illustrated in Figure 11 [222].

Molecular dynamics computer simulation has suggested that the insertion of hydrophobic nanoparticles could lead to deformation and heterogeneity of the lipid bilayer, but that this would not cause membrane leakage, while semi-hydrophilic nanoparticles appear to be energetically absorbed on the surface of the bilayer, thus, inducing their endocytosis [223]. In other theoretical or experimental studies, different nanoparticles were used to investigate the influence of hydrophobicity on the elastic properties of cell membranes, on the stability of pre-existing pores in the lipid bilayer, on membrane penetration, and, therefore, on cell function [224,225,226,227,228].

Surface functionalization of nanoparticles by modifying their surface chemistry, charge, and hydrophobicity can obviously alter their targeting efficacy and cellular uptake rates. Indeed, increasing the number of amino groups (–NH_2_), which enhances the positive surface charge, was shown to increase the internalization of nanoparticles into cells. However, the presence of –COOH functional groups, which increases the negative charge, enhances their further uptake into the endosomal compartments [229,230]. In different studies, it has also been reported that functionalized nanoparticles, such as polydopamine functionalized nanoparticle-aptamer bioconjugates, folic acid-functionalized nanoparticles, and poly(diallyldimethyl ammonium chloride)-coated gold nanorods, have better targeting efficacy and higher efficiency of internalization by cells [231,232,233].

As already mentioned, nanoparticles enter the cells through active processes because of their ability to interact with the cellular machinery. When the nanoparticles come into contact with biological fluids, such as the serum of a cell, a selective layer of proteins and other biomolecules adsorbs on their surface within a few seconds, forming the so-called corona [234], which mediates, in situ, the interactions with cells. As a consequence, one nanomaterial may cause a very different biological outcome when exposed to cells in the presence or absence of a preformed corona. More specifically, silica nanoparticles exhibited stronger adhesion to the cell membrane and higher internalization efficiency when they were exposed to cells in the absence of serum, as compared to those in a medium containing serum, where a corona was formed. The different conditions of exposure not only affected the levels of uptake but resulted in variation in the location of the intracellular nanoparticles and their impact on the cells. It is important to note that certain studies showed that, after just 1-h of exposure, a corona of very different nature can be formed on the nanoparticles exposed to cells in the absence of serum. This different outcome was attributed to the different adhesion and surface properties under the two conditions [234]. The protein adsorption capability is also affected by the nanoparticle properties. For example, both surface roughening and hydrophobic modification of the nanoparticles enhance the protein adsorption capacity and affect the cellular uptake performance; however, the relative importance of the two contributions depends on the cell type [235,236].

#### 3.1.4. Role of Cell Type on Cellular Uptake

**Figure 12:**
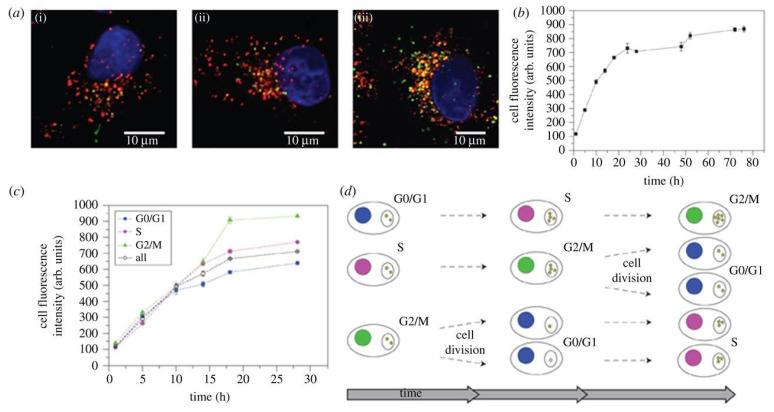
Dependence of the internalization of ~40 nm carboxylated polystyrene nanoparticles (25 μg/mL in cMEM) in A549 human lung carcinoma cells on the cell cycle phase for exposures up to 72 h. (**a**): Confocal microscopy images after cell exposure to nanoparticles for (**i**) 5, (**ii**) 24, and (**iii**) 72 h show the nanoparticle accumulation in the lysosomes. Blue: cell nuclei (DAPI); red: lysosomes (LAMP1 antibody); green: nanoparticles. (**b**): Mean cell fluorescence intensity as acquired by flow cytometry as a function of time. (**c**): Mean fluorescence intensities as a function of time of A549 cells in the G0/G1, S and G2/M phases, respectively. (**d**) Schematic of populations of the G0/G1, S, and G2/M phases by cells and consequences for cellular NP content as a function of time (Adapted with permission from ref. [189] and ref. [237]. Copyright 2012 Nature Publishing Group and 2013 Royal Society Publishing.

The role of cell cycle in the cellular uptake and dilution of nanoparticles in a cell population has also been investigated, as illustrated in Figure 12 [189,237]. It has been observed that the cellular uptake of nanoparticles is also influenced by the cell cycle phase Although more-or-less similar rates of nanoparticle internalization by the cells were observed for different phases of the cell cycle, after 24 h, the concentration of nanoparticles in the cells could be ranked according to the different phases, as follows: G2/M > S > G0/G1, where G0 is the resting phase, G1 is the phase during which the cell increases its size, S the phase when the cell synthesizes DNA, G2 the one it synthesizes proteins to prepare for cell division, and M the phase when the cell divides and the two daughter cells enter the G1 phase. During cell division, nanoparticles that are internalized by the cells are not exported but are split between daughter cells. Thus, it was indicated that, in a cell population, the dose of internalized nanoparticles in each cell varied as the cell advanced through the cell cycle.

In general, nanoparticles, due to their ability to be endocytosed, cause completely different cell responses from bulk surfaces of the same material. In spite of what has been achieved so far in the materials and nanotechnology fields, a complete understanding from a biological point of view is still missing. In this context, emerging technologies such as omics, high-throughput screening systems, and organ-on-a-chip technologies, in synergy with computational approaches, should enable, not only the analysis and documentation of large amounts of data, but also the decoding of nano–cell interactions [178,238].

### 3.2. Optical and Electronic Properties and Catalytic Activity

**Figure 13:**
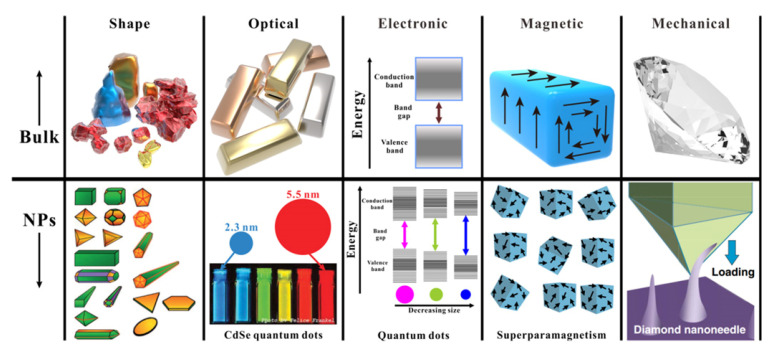
Schematic comparison between bulk materials and nanomaterials: nanoparticles with varying mechanical, electronic, optical, and magnetic properties, due to their different size and shape [239].

The nanometer size of manufactured nanomaterials results in very interesting and very important size effects that affect their chemical, structural, thermal, spectroscopic, electronic, magnetic, and mechanical properties; these effects are on top of any possible influence of the chemistry of their bulk crystals. This is schematically illustrated in Figure 13 [239].

Moreover, a single manufactured nanomaterial (MNM) may function differently in various systems; thus, it is important to carefully design MNMs to develop devices with enhanced performance, safety, and stability for both humans and the environment. While material chemistry and nanomaterial size and shape play a significant role in the core properties of an inorganic nanoparticle, the selection of ligand molecules, which functionalize the surface of the MNMs, is of great significance for their colloidal function and stability [240]. In this part of the work, a series of studies on the key properties of MNMs affecting the functionalities relative to applications are discussed; the emphasis is on the electronic and optical properties and the catalytic activity of materials and devices. It is noted that most functionalities of this type are correlated with the MNM’s key properties.

#### 3.2.1. Catalytic Properties

Generally, the catalytic properties of nanomaterials are far superior compared to bulk materials. ZnO nanomaterials are characterized as possible candidates for transistors, solar cells, light-emitting diodes, sensors, nano-lasers, photocatalysts, and antimicrobial agents because of their good stability, low cost, high excitation binding energy (60 meV), wide band gap (3.37 eV), and widespread availability [241]. Moreover, ZnO properties could be enhanced by doping with elements such as Mg [242], Al [243], and Cu [244,245]. In particular, Cu-doping of ZnO nanomaterials improved the optical properties by creating impurity levels localized in the optical energy band gap [246]. Furthermore, the optical energy band gap is reduced when the average size of the crystallites decreases, because Cu ions are incorporated into the ZnO structure [247]. The catalytic activity of ZnO nanomaterials in the presence of light has been widely investigated for environmental applications (e.g., purification), and this was found to depend on the oxygen vacancies and the morphology of ZnO. Specifically, the photocatalytic performance of ZnO nanodisks for the decomposition of methylene blue dye was enhanced because of the higher population of (0001) crystal plane structures [248]. Furthermore, ZnO nanorods with a cone of small aspect ratio are more effective in the photocatalytic degradation of organic pollutants than ZnO nanorods with a cone of large aspect ratio and ZnO microrods that are short-and-fat [249]. Moreover, ZnO nanosheets and nanoflowers demonstrated a much higher photocatalytic activity for the degradation of methyl orange than ZnO nanospheres [250]. The decomposition of volatile organic compounds, such as butane, was investigated, taking advantage of the photocatalytic activity of ZnO nanomaterials over multi-channel porous alumina ceramic membranes coated with ZnO nanoparticles, nanorods, and nanowires; the activity depended strongly on the shape of the nanomaterial used [251]. It was reported that ZnO nanowires showed a higher catalytic activity than ZnO nanoparticles or nanorods and, most importantly, the process did not result in unwanted byproducts such as propane, acetaldehyde, and acetylene. Moreover, better carbon balance and selectivity towards carbon oxides were obtained with the ZnO nanowires and nanorods than with nanoparticles. ZnO structure, shape, and crystallite size are also important parameters for their antimicrobial performance [252]. ZnO nanoflowers showed enhanced photocatalytic activity in Escherichia coli and Staphylococcus aureus inactivation compared to ZnO nanorods or nanospheres.

The optimization of catalytic performance requires the adjustment of both catalytic activity and mass transfer. Various bioinspired inner-mobile multifunctional ZnO/CdS heterostructures have been synthesized, with their artificial cilia mimicking natural ciliary motion (assisted by external magnetic fields and internal magnetism). Such a synthesis resulted in a three-times better photocatalytic performance of mobile arrays compared to static arrays [253].

#### 3.2.2. Sensing Behavior

A bioelectrochemical sensing interface can be engineered with functional nanomaterials, so as to develop novel electro-chemical biosensors with enhanced performance in terms of simplicity, sensitivity, selectivity, and stability [254]. It should be noted that the use of functional nanomaterials for the development of novel biosensors takes advantage of nanomaterial properties such as conductivity, high surface area, and improved catalytic activity; and such properties depend on the size and shape of the nanomaterials, which control, e.g., the optical properties of metal nanoparticles [255], the electrical conductivity of the carbon nanomaterials [256], as well as the electrocatalytic properties of nano-carbons and metal nanoparticles [257], etc.

Carbon nanomaterials (CNMs) exhibit unique electrical, optical, thermal, mechanical, and chemical properties and are, thus, extensively applied in photovoltaic, electronic, optoelectronic, and sensing devices. A more recent application of CNMs in the biosensing field is their use in the area of electrochemical aptasensors (ECASs) [258]. ECASs use aptamers (short single-stranded oligonucleotides of DNA or RNA), selected through a systematic evolution of ligands using an exponential enrichment technique (from a random oligonucleotide library), as recognition elements and exhibit the advantages of low cost, simple operation, fast response, and high sensitivity. A concentration- or activity-related electrochemical signal is produced by the transducers as a result of the recognition reaction. Clinical diagnosis via DNA analysis, immunoassay, or enzymatic sensing, as well as for environmental monitoring, including ocean and atmospheric pollutants, are the main detection strategies [258].

The use of carbon nanomaterials significantly increases the detection efficiency of sensors, in terms of sensitivity, selectivity, and stability, and has become one of the current development strategies for ECASs-based sensing platforms. The excellent electrical conductivity and high specific surface area of the CNMs allow them to function as electronic conductive matrices and immobilization platforms for the aptamers [258,259]. These properties depend on the atomic structures of the different CNMs, such as graphene, graphene oxide, carbon nanotubes, etc., as well as on their interactions with other nanomaterials, such as chitosan, silica, or gold nanoparticles. In particular, carbon nanotubes (CNTs) are commonly used as catalyst carriers or backing layers. CNTs demonstrate an enhanced electro-catalytic activity and a very large surface area to volume ratio, with multi-walled carbon nanotubes (MWCNTs) being used more often in ECASs applications than single-walled carbon nanotubes (SWCNTs). Moreover, combining CNTs with other nanomaterials (e.g., gold nanoparticles, reduced graphene oxide, dendrimers, chitosan, etc.) can further enhance the carrier content and stability of enzymes and proteins. Graphene, graphene oxide, and reduced graphene oxide have also been utilized in ECASs [260,261], with the main differences in this application originating from their significantly different electrical conductivities; the effectiveness of these three types of CNMs follows their ranking of conductivities, with graphene being preferable for ECASs development, followed by reduced graphene oxide and, then, graphene oxide.

Improved device performance and notably enhanced electrical properties were reported when SWCNTs were assembled into aligned arrays with full surface coverage (via the Langmuir–Schaefer method). The intrinsic mobility of the CNTs was preserved for a semiconducting nanotube purity of 99% and full surface coverage and, thus, for high packing density [262].

The use of carbon nanomaterials to construct functional composites was reviewed [263], and effective methods were presented to achieve light harvesting and conversion, effective phonon transport along a particular direction, and rapid ion and electron motion in structural electrodes through the chemical grafting of functional groups to improve their reactivity and thermal stability [263]. Moreover, novel optical-triggered graphene-based actuators were fabricated with a bilayer structure including chitosan and polyethylene (PE) over a large area [264]. The graphene nanosheets played the role of a connecting bridge between light and the conversion of light energy at the nanoscale.

The hybridization of different types of carbon nanomaterials has been utilized to enable many different properties and performances beyond that of the individual nanomaterials, for example in electrochemical or analytical devices. Hybrid nanomaterial systems are, in principle, designed to develop more efficient sensors. Each nanomaterial exhibits its own advantages for various applications; thus, it is important to involve synergies due to the presence of the different nanomaterials, so as to complement each other in the hybrid system [265,266]. For example, graphene–inorganics composites that take advantage of the properties of both graphene and the inorganic elements (e.g., gold nanoparticles) enable even higher active surface areas and enhanced rates of electron transfer. Thus, functional hybrids are developed based on graphene nanosheets, in order to take advantage of the electrical, optical, and catalytic properties of graphene and enhance its performance in analytical chemistry and electrochemistry [256].

MWCNT-modified electrodes have been used to investigate the electrochemical oxidation of nicotinamide adenine dinucleotide (NADH) and to elucidate their respective mechanisms of oxidation [257]; the study compared the behavior with cases when boron-doped diamond and glassy carbon electrodes were used, as well as with cases when edge plane and basal pyrolytic graphite electrodes were utilized, which allowed the reactive sites of carbon nanotubes to be deduced. It was concluded that electron transfer was more facile with samples containing a higher proportion of edge plane defects, compared to basal plane graphite electrodes. It was, thus, indicated that electroanalytical sensors with carbon-based electrodes should optimally possess a large proportion of edge plane sites, for achieving the best detection limits, whereas edge plane pyrolytic graphite electrodes can conveniently replace CNT-modified electrodes for routine sensing of NADH, due to their simple preparation process, low detection limit, low susceptibility to fouling of the electrode, and insensitivity to interference from ascorbic acid. It was demonstrated that an electrode produced fully of edge plane graphite (disc of pyrolytic graphite with the disc surface facing parallel with the edge plane) displayed high levels of electro-catalytic activity for different electroanalytical tasks, including gas sensing [267] and thiol oxidation [268].

Carbon nanotubes exhibit a quantum electron confinement normal to the nanotube axis, thus, being able to transport electrons over long lengths [269]. They have great potential as biomolecule immobilization platforms. According to some studies, CNTs/polymer nanostructured composites developed on electrodes can improve the analytical performance of amperometric biosensors [270,271]. Such composites display percolation behavior, by remarkably enhancing the electrode conductivity. Moreover, the CNTs thermal and electrical conductivity and their electrocatalytic activity can be modified by doping of the CNTs with elements such as K, B, Ce, N, Si, P, etc. [272,273].

Furthermore, multifunctional CNTs offer routes towards the production of smart and high-performance sensors, logic gates, and similar optoelectronic devices [274]. By combining CNTs with photochromic molecules, and in particular by decorating them, reversible changes in the geometrical structure, the electronic properties, and the nanoscale mechanics triggered by light can be achieved [274]. As a result, there is control of the local variation in the optical, electrostatic, and mechanical environment with light illumination. For example, azobenzenes blended with CNTs and polymers are used to form nanocomposites possessing light-induced conductance switching properties; such nanocomposites are good candidates for electro-optical memories, smart packaging, and smart window applications [275]. A graphene/azobenzene/Au heterostructure switch was found to further induce the reversible modification of the electrical and quantum properties of the Dirac fermions of graphene [276]. Furthermore, a hybrid system of chemically grafted spiropyrans to CNTs was utilized to regulate horseradish peroxidase (HRP) activity via light illumination. This resulted in enhancement of the catalytic activity of HRP and was used as a label-free colorimetric lysozyme assay with a detection limit of 30 nM. This high selectivity approach can be applied to regulate the activity of other natural proteins using light [277].

#### 3.2.3. Optoelectronic Properties

Certain nanomaterials are used as biomolecular labels because they exhibit unique optical properties. They amplify biorecognition signals and enhance the biosensor sensitivity [269]. Various nanoparticles, including metal, oxide, or semiconductor nanoparticles and their composites, have been widely used in the fields of biosensors and electrochemical sensors [278]. The majority of the nanoparticles possess a high isoelectric point (IEP), favoring electrostatic protein adsorption with low IEP. Thus, they are promising supports for protein immobilization. A cholesterol biosensor consists of an interfacial layer of gold nanoparticles, which is used for immobilizing cholesterol oxidase on gold electrode surfaces. Here, gold nanoparticles provided an environment for the enhanced electrocatalytic activity of cholesterol oxidase and, thus, improved the stability of the biosensor [279]. The gold nanoparticles were found to favor the analytical performance of the cholesterol biosensors; this was attributed to the biocompatibility of the gold nanoparticle-based immobilization matrices, to assist proteins in retaining their biological activity for long periods and, thus, improve the stability of the biosensor [269]. The enhancement of the sensitivity and selectivity of the biosensor was mainly due to the electrocatalytic activity of the gold nanoparticles; gold nanoparticles improved the conductivity of the electrodes and facilitated the electron transfer between the electrode and the enzyme redox center. Gold nanoparticles on flat electrode surfaces may also partially penetrate the enzyme matrix and, thus, come closer to the enzyme redox center, which further aids the electron transfer pathway.

Interesting nanomaterials include the helical carbon nanofibers (CNFs), with excellent optical, electromagnetic, and mechanical properties, due to their unique spiral structure; aiming at applications such as microwave absorbing materials and electrode materials [280]. To improve the optical, physical, mechanical, and chemical properties of CNFs, more functional building blocks were incorporated, to form CNF-based composites. An example is the in situ synthesized mesoporous N-CNFs containing graphitic-C_3_N_4_ (g-C_3_N_4_), in which the strong coupling between the components of the CNFs enabled the final material to have an efficient optical storage performance, improved charge separation, and multi-dimensional electron transport path; thus, improving the performance of hydrogenation production, as well as the performance in photocatalytic and optoelectronic applications [281].

Another application of gold nanoparticles in the medical field is in cardiac tissue engineering, due to their controlled geometrical, surface, chemical, and optical properties [282]. Additionally, gold nanoparticles enhance the electrical conductivity of nanocomposites. High electrical conductivity, acceptable biocompatibility, the capability for surface modification, nanotopography, and innate optical properties make this nanoparticle type a desirable nanostructure for cardiac scaffolds [283].

Metal oxide nanoparticles are able to achieve low detection limits in analysis, due to their electron transfer [284,285]. Moreover, the capability for enhanced adsorption of the biomolecules leads to high biosensor stability. Cerium oxide (CeO_2_), iron oxide (Fe_3_O_4_), zinc oxide (ZnO), and titanium oxide (TiO_2_) nanoparticles have been exploited for improving sensor performance [286,287,288]. Gold nanoparticles exhibit outstanding optical properties as well; this is due to the surface plasmon resonance (SPR) phenomenon, when the light interacts with the collective oscillations of electrons on the gold nanoparticle surface at a certain light wavelength [269]. This depends on the shape, size, and state of aggregation of the gold nanoparticles. An important application is in the field of detection assays, where an alteration of the light extinction that results from the aggregation of gold nanoparticles upon analyte addition is used as the optical signal [289].

**Figure 14:**
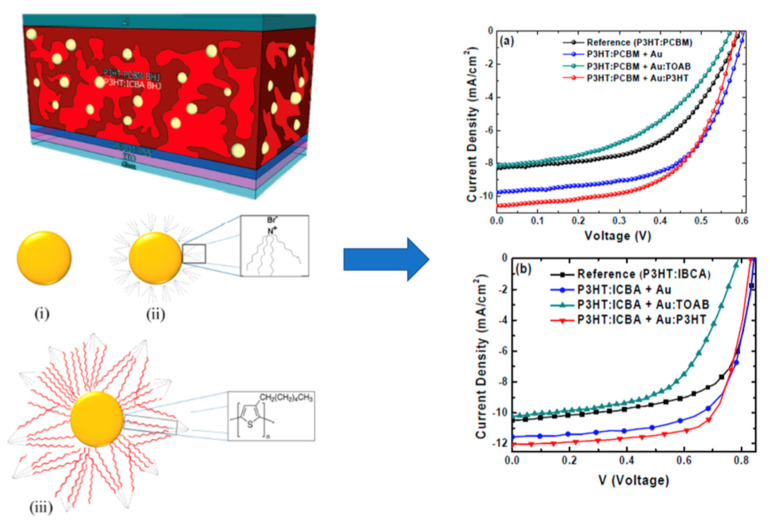
Schematic representation of a bulk heterojunction organic photovoltaic cell with three kinds of nanoparticles within the active layer: (**i**) bare, (**ii**) TOAB-functionalized, and (**iii**) P3HT-functionalized. J−V curves of the devices with configurations (**a**) ITO/PEDOT:PSS/P3HT:PCBM/Al and (**b**) ITO/PEDOT:PSS/P3HT:ICBA/Ca/Al, respectively (Reprinted with permission from ref. [240], Copyright 2019 American Chemical Society) with the original data from Ref. [292], Copyright 2015 American Chemical Society). Nomenclature: ITO: indium tin oxide; PEDOT: poly(3,4-ethylenedioxythiophene); PSS: poly(styrene sulfonate); P3HT: poly(3-hexylthiophene-2,5-diyl); PCBM: [6,6]-phenyl-C_61_-butyric acid methyl ester; ICBA: indene-C60 bisadduct.

The incorporation of nanoparticles into various building blocks within the solar cell architecture, in order to enhance photovoltaic performance and stability, has also been reported [290]. It was observed that the conversion efficiency of solar cells with silicon nanocrystals was 5.3-times higher than one with only titania (TiO_2_) particles, contributing to further light absorption and, thus, to an improvement of the conversion efficiency. Further incorporation of nanoparticles such as Ag and Au, produced via laser ablation in liquids, into the active/hole transport layer interface of P3HT:PCBM bulk heterojunction solar cells was reported to lead to an enhanced conversion efficiency [291]. The role of ligand coatings on nanoparticles in the photovoltaic performance has also been discussed, as illustrated in Figure 14 [292].

The chemical, optical, electrical, thermal, and magnetic properties of magnetic nanoparticles can also be exploited in various steps of analytical processes, including sample treatment, chromatographic techniques, and detection [293]. Iron oxides (Fe_2_O_3_ and Fe_3_O_4_) and their corresponding ferrites (e.g., MnFe_2_O_4_ or CoFe_2_O_4_) are commonly utilized because of their biological compatibility, the simple preparation processes, and high magnetic moment relative to other nanoparticles based on metals and alloys (e.g., Mn_3_O_4_, Co, Ni, FePt), which exhibit rapid oxidation in air and/or potential cytotoxicity. Magnetic nanoparticles can be modified with inorganic, organic, or biochemical compounds to improve their physicochemical behavior. For example, hybrid magnetic nanoparticles are developed by the combination of Fe_3_O_4_ nanoparticles and carbon, metallic, polymeric, or silica nanoparticles for the manufacturing of electrodes, thus improving their electrocatalytic properties, among others [294]. Such electrodes are advantageous, due to their large surface area, low resistance to electronic transmission, and ability to adsorb (bio)chemical analytes, which make them useful in electrochemical systems. The main advantages of magnetic nanoparticles in this area are the increase of electrocatalytic activity, the minimization of deterioration of the electrode surfaces, and the simplification of the immobilization process [293].

Last, we would like to point out that in this mini review we have mostly discussed the behavior of single nanoparticles, and not nanoparticle assemblies [295]. The formation of the latter is mostly induced by the very high surface energy of the nanoparticles, because of their high specific surface area; this provides the driving force for the spontaneous aggregation of the nanoparticles, which would decrease the Gibbs free energy of the system and would lead to large assemblies. In these cases, the performance of the nanoparticles for various applications will be based on the coupling of, and cooperation among, individual nanoparticles, rather than on their individual properties; this collective behavior would, of course, depend on the interparticle interactions that would determine their structural arrangement in space [239]. Such nanoparticle assemblies may lead to a plethora of practical applications, such as sensing, energy storage, strong materials, catalysis, therapies, etc. Moreover, introducing different nanoparticles into a superlattice can lead to substitutional doping when the size of the two types of nanoparticles are similar, in an analogy to the classical doping process where atomic impurities are intentionally added to a host material to significantly modify its properties; the electronic properties of such doped superlattices are significantly influenced by the presence and density of the nanoparticle dopants, leading to highly tunable nanomaterials [296].

## 4. Concluding Remarks

Nanotechnology, which deals with the understanding and control of matter in dimensions between about 1 and 100 nanometers and where unique phenomena allow new applications, has enabled the development of a variety of nanomaterials with unique properties, aimed at various applications. Thus, it becomes apparent that the interaction of nanomaterials with their environment is governed by different mechanisms and leads to new responses.

To summarize the main points of this literature review, the key parameters of manufactured nanomaterials that play an important role for each of the functionalities are outlined below:

The **dispersion ability** of the nanomaterials is a key issue affecting their behavior. Nanoparticles form, in general, aggregates and/or agglomerates in water or other aqueous media; SiO_2_ nanoparticles are the only exception, where the primary particle size is detected in certain cases. The dispersion ability is affected by the particle chemical composition, the existence of an appropriate surface coating, the surface charge, as well as by the dispersion media, whereas it depends only weakly on their shape and crystallinity. The particle size is not that crucial in determining dispersibility, except when nanoparticles and particles with radii larger than 300–400 nm are compared, because of the influence of gravity. Apart from the nanoparticles themselves, the presence of organic moieties in the solution (e.g., proteins), the solution pH and its ionic strength affect dispersibility.

The **hydrophobicity/hydrophilicity** of nanoparticles and other manufactured nanomaterials depends on their chemical characteristics (chemistry, surface charge) and their surface coating (characteristics, surface reactivity and stability). Besides the effects of hydrophobicity/hydrophilicity on the dispersibility, with hydrophilic nanoparticles being more easily dispersed in aqueous media than hydrophobic ones, nanoparticle hydrophobicity/hydrophilicity is also very important for their biocompatibility. Hydrophobic nanoparticles can be rendered hydrophilic by appropriate modification of their surface using surfactants or various hydrophilic polymers.

**Solubility/dissolution** of the nanoparticles implicates the release of ions from the nanomaterials into the solution. It is a function of the nanoparticle characteristics, such as chemistry, composition, size and surface area, surface coating, and crystallinity. It is also affected by the pH and the temperature of the solution. The dissolution of nanoparticles affects their antimicrobial activity and biocompatibility.

The physicochemical properties of nanoparticles, such as size, shape, and surface properties, control the internalization pathways, thus, playing a pivotal role in **cellular uptake**. In biomedical applications of nanoparticles, their coating modification has been shown to affect the modulation of their cellular internalization. It is important to take into consideration the possible sedimentation of large and/or dense particles and their diffusion velocities when in vitro studies are performed utilizing large and/or heavy nanomaterials. Moreover, the formation of a protein corona on the nanomaterial surface and its composition play an important role in the possible cellular uptake.

Individual nanomaterials can play various roles in devices in the field of biosensing. Depending on the desired application, their main key parameters should be designed and tuned carefully, whereas composite systems are frequently used to enhance the performance with regards to detection, stability, and duration. **The optical and electronic properties and the catalytic activity of the nanomaterials** are functionalities that depend on their size and shape, whereas the organization of the individual nanomaterials in a hybrid affects the general performance of the various devices.

All of the above findings are illustrated in the two following Tables. Table 1 demonstrates how the three functionalities that have been discussed are affected by the main key parameters, whereas Table 2 shows how the key parameters influence the final properties (optical, electronic, and catalytic properties and the cell uptake). The key parameters discussed have been grouped into six categories, i.e., as geometrical, chemical, crystallinity, morphological, coating related, and test medium related parameters. In the tables, we have introduced the notation of two stars (**) to illustrate that a parameter is a ‘priority’; i.e., it significantly determines a particular functionality/property, and the notation of one star (*) to illustrate that a parameter is ‘of importance’; i.e., it is important but it does not determine the behavior by itself. According to Table 1, it is clear that key parameters like the chemical composition, the existence of a surface coating, and the test medium are of utmost importance related to all functionalities, whereas the significance of the others should be deduced case by case. As far as Table 2 is concerned, it is the size, the shape, the chemical composition, and the surface charge of nanoparticles that influence, in general, all properties.

One should also point out that an inter-relation exists between the parameters and the functionalities, and this significantly affects the final properties and, thus, the applications in which the nanomaterials are used. Moreover, it is noted that the Nanotechnology Characterization Laboratory (NCL) at the National Cancer Institute USA, which has assessed more than one hundred and thirty different types of nanomaterials, including metal oxides, fullerenes, liposomes, dendrimers, polymers, quantum dots, and gold colloids, came to the conclusion that hydrophobicity (which is a ‘functionality’), and size and surface charge (which are ‘key parameters’) are the main factors that influence nanomaterial biocompatibility [297].

## 5. Challenges and Prospects

In order to advance knowledge in the area of the physicochemical properties/functionalities of nanoparticles, on how these are determined by their key parameters, and, more importantly, on how these influence their behavior and their potential to induce, or not induce, toxicity to both humans and the environment, as well as their ultimate fate more focused research is still needed in this area. Despite the plethora of related works, there are still many open challenges with regards to the interrelationships between the physicochemical main key parameters of nanoparticles and their functionalities, which are considered as very important aspects for enhancing their safety early on in the design process.

Such challenges include:

- Understanding the interdependence between the bulk properties of the materials (i.e., in their pristine state) versus the respective properties when the materials exist in nanodimensions within a particular medium, i.e., dispersed in a biological fluid
- Developing different production/manufacturing routes and different residues
- Understanding and, possibly, modifying different experimental conditions, e.g., instruments, protocols, in vitro versus in vivo methodologies
- Improving the measuring tools for site-specific or local assessment of nanomaterials, e.g., high resolution imaging, 3D reconstruction, data acquisition processes

To improve the design of a nanomaterial, one needs to consider the use of innovative tools to probe the dynamic biophysicochemical interactions. The adoption and optimization of both theoretical and experimental characterization methods, which are traditionally utilized for characterizing the properties of bulk materials, for studies of the environment surrounding nanomaterials and the resulting interfaces is mandatory. This will also be helped by simple and widely accessible laboratory equipment.

Research is, therefore, needed at the interface of different disciplines, such as engineering, physics, chemistry, biology, and medicine. This research should aim at the advanced chemical synthesis of new nanostructures with precisely defined biophysicochemical characteristics and properties, at the development of nanostructures that will replace biological structures, and at addressing the knowledge gaps concerning the possible health and safety effects of exposure to manufactured nanomaterials. Such research will be able to give prominence to nanomedicine as a promising stakeholder in the field of diagnosis, imaging, treatment, therapeutics, and regenerative medicine.

## Figures and Tables

**Table 1:** How the key parameters of nanomaterials affect performance.

		PERFORMANCE		
KEY PARAMETERS		Dispersion	Solubility/Dissolution	Hydrophobicity/Hydrophilicity
Geometrical	Particle Size (e.g., hydrodynamic radius and polydispersity index)	*	**	*
	Shape	*	**	*
	Aspect Ratio	*	*	
Chemical	Composition	**	**	**
	Surface charge/ζ potential	**	*	*
Crystallinity	Crystal structure/Crystallinity	*		
Morphological	Topology (e.g., core shell, etc.)			
	Porosity		*	
	Surface area	*	*	*
	Roughness		*	*
Coating	Chemistry, Thickness, Topology	**		*
	Surface Coating Stability		**	**
	Surface reactivity		**	**
Test Medium	Kind	**	**	**
	pH	**	**	**
	Ionic Strength	**		*

**: a key parameter designated as ‘a priority’ (see text); *: a key parameter designated as ‘of importance’ (see text).

**Table 2:** How the key parameters of nanomaterials affect their applications.

		APPLICATIONS			
KEY PARAMETERS		Cellular Uptake	Optical Properties	Electronic Properties	Catalytic Activity/Biorecognition
Geometrical	Particle Size (e.g., hydrodynamic radius and polydispersity index)	**	**	**	**
	Shape	**	**	**	**
	Aspect Ratio	**	*	*	*
Chemical	Composition	**	*	**	**
	Surface charge/ζ potential	**	**	**	
Crystallinity	Crystal structure/Crystallinity		*	*	*
Morphological	Topology (e.g., core shell, etc.)				
	Porosity				
	Surface area	*	*	*	*
	Roughness	*			
Coating	Chemistry, Thickness, Topology	**	*	*	*
	Surface Coating Stability		*	*	
	Surface reactivity	*	*	*	*
Test Medium	Kind	**	*		
	pH	*			
	Ionic Strength	*			

**: a key parameter designated as ‘a priority’ (see text); *: a key parameter designated as ‘of importance’ (see text).

## Data Availability

Data presented in this review article are available from the authors of the cited publications.
